# Supplementary material for: RBD-specific antibody responses after two doses of BBIBP-CorV (Sinopharm, Beijing CNBG) vaccine
Source: BMC Infect Dis. 2022 Jan 24;22:87. doi: 10.1186/s12879-022-07069-z (PMC8785690; doi:10.1186/s12879-022-07069-z)
Supplement: Supplementary file 1 — Additional file 1: Figure S1. Effects of the age and the sex of the subject, and the time-period between the vaccination and the measurement on the probability of the lack of RBD-specific antibody production (titre below 1) after two doses of the Sinopharm vaccine using logistic regression model. 90% credible interval is shown for males, 28 days post second dose. Figure S2. Effects of the age and the sex of the subject on the probability of the lack of RBD-specific antibody production (titre below 1) after two doses of the Pfizer/BioNTech vaccine using logistic regression model. 90% credible interval is shown for males, 28 days post second dose. Figure S3. Sinopharm vaccine model, MCMC diagnostics: density plot for the hurdle-lognormal model. Figure S4. Sinopharm vaccine model, MCMC diagnostics: density plot for the logistic model. Figure S5. Sinopharm vaccine model, MCMC diagnostics: trace plot for the hurdle-lognormal model. Figure S6. Sinopharm vaccine model, MCMC diagnostics: trace plot for the logistic model. Figure S7. Sinopharm vaccine model, MCMC diagnostics: autocorrelation function for the hurdle-lognormal model. Figure S8. Sinopharm vaccine model, MCMC diagnostics: autocorrelation function for the logistic model. Figure S9. Sinopharm vaccine model, MCMC diagnostics: posterior predictive check for the hurdle-lognormal model. Figure S10. Sinopharm vaccine model, MCMC diagnostics: posterior predictive check for the logistic model. Figure S11. Pfizer/BioNTech vaccine model, MCMC diagnostics: density plot for the hurdle-lognormal model. Figure S12. Pfizer/BioNTech vaccine model, MCMC diagnostics: density plot for the logistic model. Figure S13. Pfizer/BioNTech vaccine model, MCMC diagnostics: trace plot for the hurdle-lognormal model. Figure S14. Pfizer/BioNTech vaccine model, MCMC diagnostics: trace plot for the logistic model. Figure S15. Pfizer/BioNTech vaccine model, MCMC diagnostics: autocorrelation function for the hurdle-lognormal model. Figure S16. Pfi [file 12879_2022_7069_MOESM1_ESM.zip › 12879_2022_7069_MOESM1_ESM/Sinopharm_study_paper_BMCID_2_Additional.docx]

Additional material

# Analysis with logistic regression

Data were secondarily analysed with logistic regression as a sensitivity analysis to ensure that results are robust with respect to the chosen analytical method. Results are shown on Figure S1 (Sinopharm vaccine) and Figure S2 (Pfizer/BioNTech vaccine). These completely corroborate the results obtained with the main analytical method.

# Additional diagnostics for MCMC

Routine diagnostics were carried out for all models estimated with MCMC: trace plots, density plots, autocorrelation functions and posterior predictive checks were examined. These are provided both for the Sinopharm vaccine models (Figures S3 to S10) and for the Pfizer/BioNTech vaccine models (Figures S11 to S18).

# Additional figure captions

Figure S1. Effects of the age and the sex of the subject, and the time-period between the vaccination and the measurement on the probability of the lack of RBD-specific antibody production (titre below 1) after two doses of the Sinopharm vaccine using logistic regression model. 90% credible interval is shown for males, 28 days post second dose.

Figure S2. Effects of the age and the sex of the subject on the probability of the lack of RBD-specific antibody production (titre below 1) after two doses of the Pfizer/BioNTech vaccine using logistic regression model. 90% credible interval is shown for males, 28 days post second dose.

Figure S3. Sinopharm vaccine model, MCMC diagnostics: density plot for the hurdle-lognormal model.

Figure S4. Sinopharm vaccine model, MCMC diagnostics: density plot for the logistic model.

Figure S5. Sinopharm vaccine model, MCMC diagnostics: trace plot for the hurdle-lognormal model.

Figure S6. Sinopharm vaccine model, MCMC diagnostics: trace plot for the logistic model.

Figure S7. Sinopharm vaccine model, MCMC diagnostics: autocorrelation function for the hurdle-lognormal model.

Figure S8. Sinopharm vaccine model, MCMC diagnostics: autocorrelation function for the logistic model.

Figure S9. Sinopharm vaccine model, MCMC diagnostics: posterior predictive check for the hurdle-lognormal model.

Figure S10. Sinopharm vaccine model, MCMC diagnostics: posterior predictive check for the logistic model.

Figure S11. Pfizer/BioNTech vaccine model, MCMC diagnostics: density plot for the hurdle-lognormal model.

Figure S12. Pfizer/BioNTech vaccine model, MCMC diagnostics: density plot for the logistic model.

Figure S13. Pfizer/BioNTech vaccine model, MCMC diagnostics: trace plot for the hurdle-lognormal model.

Figure S14. Pfizer/BioNTech vaccine model, MCMC diagnostics: trace plot for the logistic model.

Figure S15. Pfizer/BioNTech vaccine model, MCMC diagnostics: autocorrelation function for the hurdle-lognormal model.

Figure S16. Pfizer/BioNTech vaccine model, MCMC diagnostics: autocorrelation function for the logistic model.

Figure S17. Pfizer/BioNTech vaccine model, MCMC diagnostics: posterior predictive check for the hurdle-lognormal model.

Figure S18. Pfizer/BioNTech vaccine model, MCMC diagnostics: posterior predictive check for the logistic model.
