# Supplementary figures and images for: RBD-specific antibody responses after two doses of BBIBP-CorV (Sinopharm, Beijing CNBG) vaccine
Source: BMC Infect Dis. 2022 Jan 24;22:87. doi: 10.1186/s12879-022-07069-z (PMC8785690; doi:10.1186/s12879-022-07069-z)

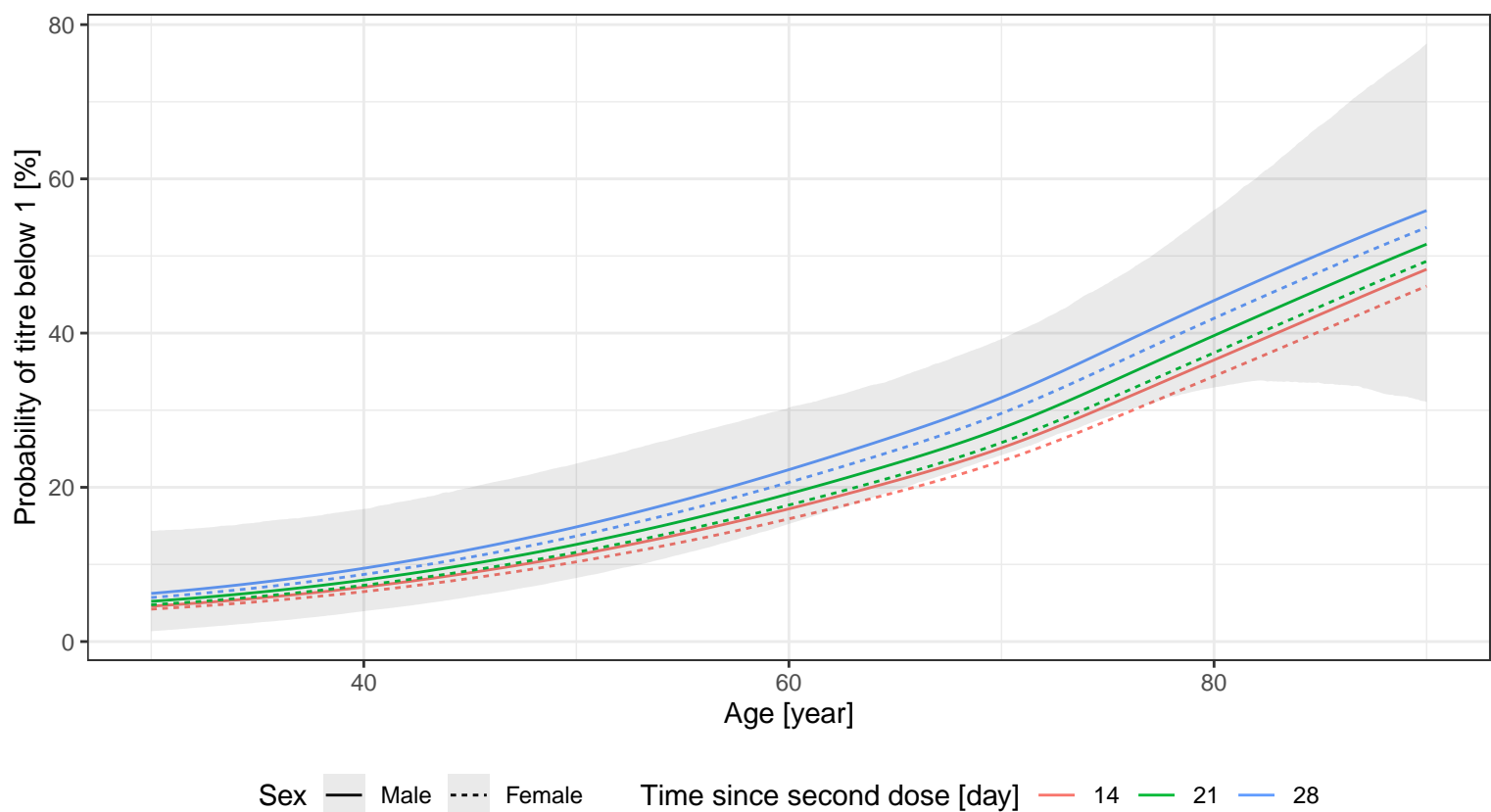

Supplement: Supplementary file 1 — Additional file 1: Figure S1. Effects of the age and the sex of the subject, and the time-period between the vaccination and the measurement on the probability of the lack of RBD-specific antibody production (titre below 1) after two doses of the Sinopharm vaccine using logistic regression model. 90% credible interval is shown for males, 28 days post second dose. Figure S2. Effects of the age and the sex of the subject on the probability of the lack of RBD-specific antibody production (titre below 1) after two doses of the Pfizer/BioNTech vaccine using logistic regression model. 90% credible interval is shown for males, 28 days post second dose. Figure S3. Sinopharm vaccine model, MCMC diagnostics: density plot for the hurdle-lognormal model. Figure S4. Sinopharm vaccine model, MCMC diagnostics: density plot for the logistic model. Figure S5. Sinopharm vaccine model, MCMC diagnostics: trace plot for the hurdle-lognormal model. Figure S6. Sinopharm vaccine model, MCMC diagnostics: trace plot for the logistic model. Figure S7. Sinopharm vaccine model, MCMC diagnostics: autocorrelation function for the hurdle-lognormal model. Figure S8. Sinopharm vaccine model, MCMC diagnostics: autocorrelation function for the logistic model. Figure S9. Sinopharm vaccine model, MCMC diagnostics: posterior predictive check for the hurdle-lognormal model. Figure S10. Sinopharm vaccine model, MCMC diagnostics: posterior predictive check for the logistic model. Figure S11. Pfizer/BioNTech vaccine model, MCMC diagnostics: density plot for the hurdle-lognormal model. Figure S12. Pfizer/BioNTech vaccine model, MCMC diagnostics: density plot for the logistic model. Figure S13. Pfizer/BioNTech vaccine model, MCMC diagnostics: trace plot for the hurdle-lognormal model. Figure S14. Pfizer/BioNTech vaccine model, MCMC diagnostics: trace plot for the logistic model. Figure S15. Pfizer/BioNTech vaccine model, MCMC diagnostics: autocorrelation function for the hurdle-lognormal model. Figure S16. Pfi [file 12879_2022_7069_MOESM1_ESM.zip › 12879_2022_7069_MOESM1_ESM/FigureS1.pdf]

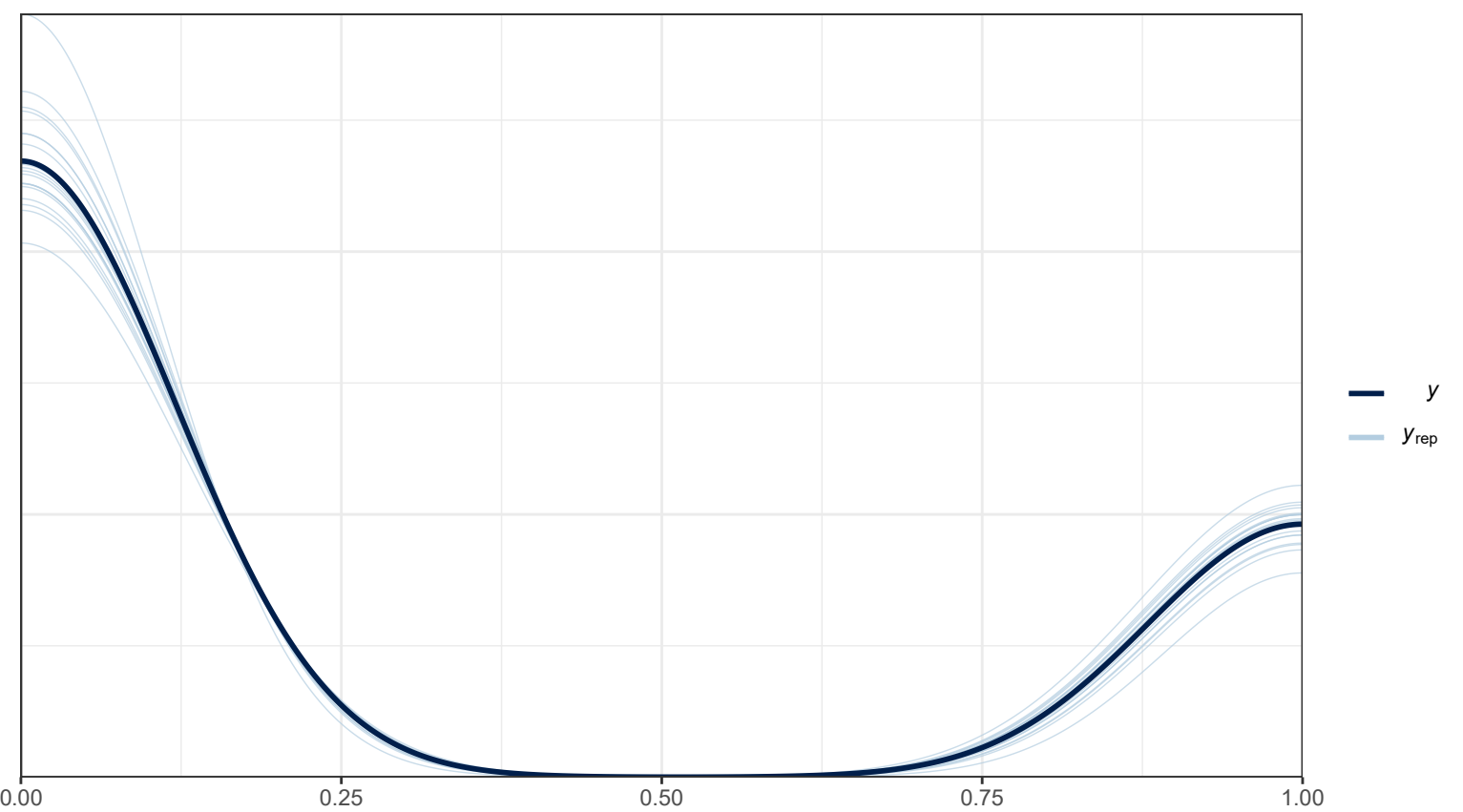

Supplement: Supplementary file 1 — Additional file 1: Figure S1. Effects of the age and the sex of the subject, and the time-period between the vaccination and the measurement on the probability of the lack of RBD-specific antibody production (titre below 1) after two doses of the Sinopharm vaccine using logistic regression model. 90% credible interval is shown for males, 28 days post second dose. Figure S2. Effects of the age and the sex of the subject on the probability of the lack of RBD-specific antibody production (titre below 1) after two doses of the Pfizer/BioNTech vaccine using logistic regression model. 90% credible interval is shown for males, 28 days post second dose. Figure S3. Sinopharm vaccine model, MCMC diagnostics: density plot for the hurdle-lognormal model. Figure S4. Sinopharm vaccine model, MCMC diagnostics: density plot for the logistic model. Figure S5. Sinopharm vaccine model, MCMC diagnostics: trace plot for the hurdle-lognormal model. Figure S6. Sinopharm vaccine model, MCMC diagnostics: trace plot for the logistic model. Figure S7. Sinopharm vaccine model, MCMC diagnostics: autocorrelation function for the hurdle-lognormal model. Figure S8. Sinopharm vaccine model, MCMC diagnostics: autocorrelation function for the logistic model. Figure S9. Sinopharm vaccine model, MCMC diagnostics: posterior predictive check for the hurdle-lognormal model. Figure S10. Sinopharm vaccine model, MCMC diagnostics: posterior predictive check for the logistic model. Figure S11. Pfizer/BioNTech vaccine model, MCMC diagnostics: density plot for the hurdle-lognormal model. Figure S12. Pfizer/BioNTech vaccine model, MCMC diagnostics: density plot for the logistic model. Figure S13. Pfizer/BioNTech vaccine model, MCMC diagnostics: trace plot for the hurdle-lognormal model. Figure S14. Pfizer/BioNTech vaccine model, MCMC diagnostics: trace plot for the logistic model. Figure S15. Pfizer/BioNTech vaccine model, MCMC diagnostics: autocorrelation function for the hurdle-lognormal model. Figure S16. Pfi [file 12879_2022_7069_MOESM1_ESM.zip › 12879_2022_7069_MOESM1_ESM/FigureS10.pdf]

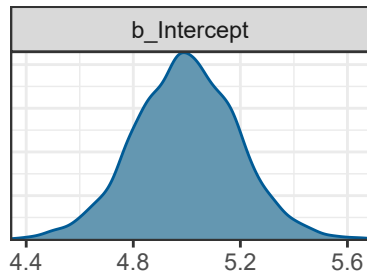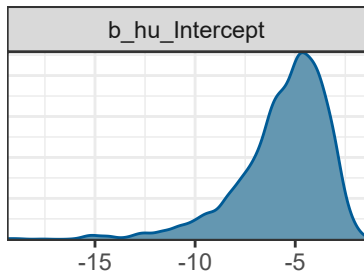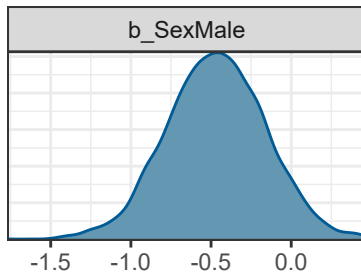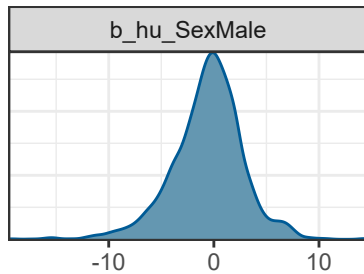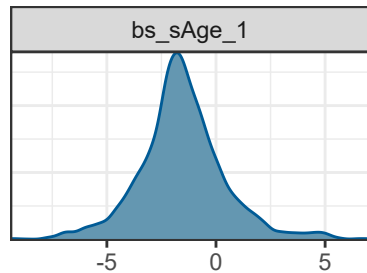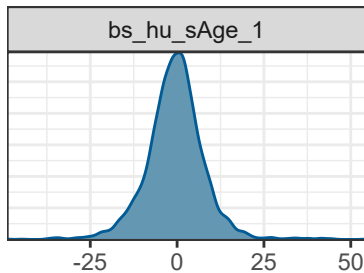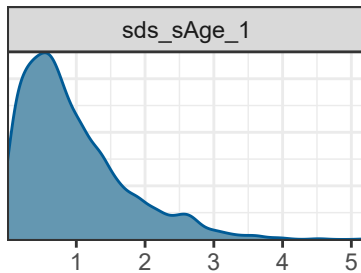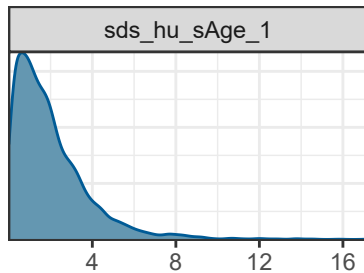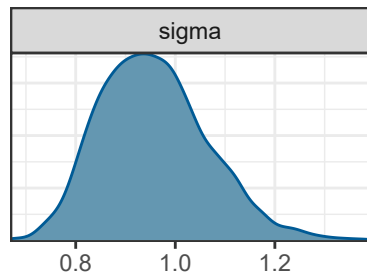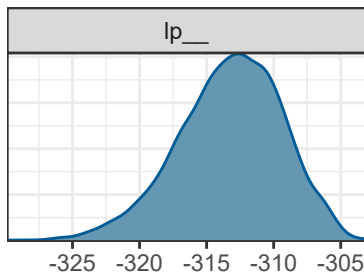

Supplement: Supplementary file 1 — Additional file 1: Figure S1. Effects of the age and the sex of the subject, and the time-period between the vaccination and the measurement on the probability of the lack of RBD-specific antibody production (titre below 1) after two doses of the Sinopharm vaccine using logistic regression model. 90% credible interval is shown for males, 28 days post second dose. Figure S2. Effects of the age and the sex of the subject on the probability of the lack of RBD-specific antibody production (titre below 1) after two doses of the Pfizer/BioNTech vaccine using logistic regression model. 90% credible interval is shown for males, 28 days post second dose. Figure S3. Sinopharm vaccine model, MCMC diagnostics: density plot for the hurdle-lognormal model. Figure S4. Sinopharm vaccine model, MCMC diagnostics: density plot for the logistic model. Figure S5. Sinopharm vaccine model, MCMC diagnostics: trace plot for the hurdle-lognormal model. Figure S6. Sinopharm vaccine model, MCMC diagnostics: trace plot for the logistic model. Figure S7. Sinopharm vaccine model, MCMC diagnostics: autocorrelation function for the hurdle-lognormal model. Figure S8. Sinopharm vaccine model, MCMC diagnostics: autocorrelation function for the logistic model. Figure S9. Sinopharm vaccine model, MCMC diagnostics: posterior predictive check for the hurdle-lognormal model. Figure S10. Sinopharm vaccine model, MCMC diagnostics: posterior predictive check for the logistic model. Figure S11. Pfizer/BioNTech vaccine model, MCMC diagnostics: density plot for the hurdle-lognormal model. Figure S12. Pfizer/BioNTech vaccine model, MCMC diagnostics: density plot for the logistic model. Figure S13. Pfizer/BioNTech vaccine model, MCMC diagnostics: trace plot for the hurdle-lognormal model. Figure S14. Pfizer/BioNTech vaccine model, MCMC diagnostics: trace plot for the logistic model. Figure S15. Pfizer/BioNTech vaccine model, MCMC diagnostics: autocorrelation function for the hurdle-lognormal model. Figure S16. Pfi [file 12879_2022_7069_MOESM1_ESM.zip › 12879_2022_7069_MOESM1_ESM/FigureS11.pdf]

b\_Intercept

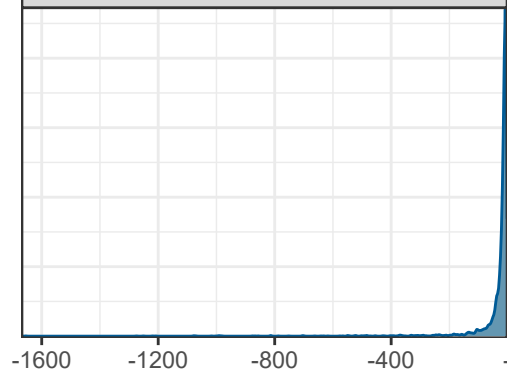

b\_SexMale

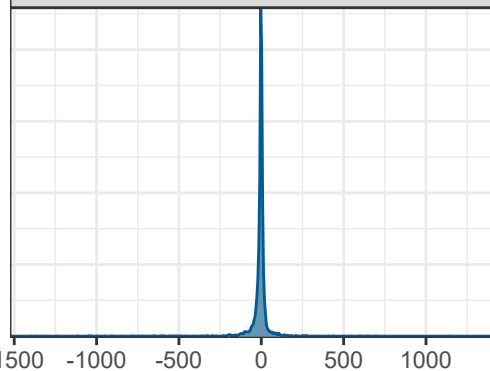

bs\_sAge\_1

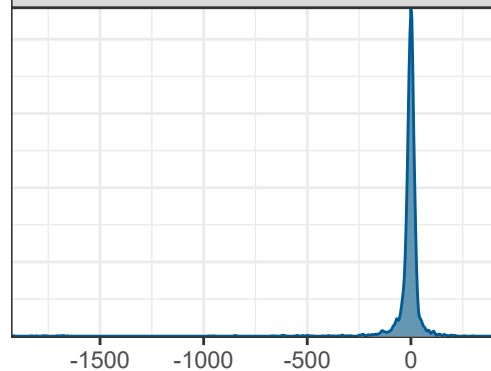

sds\_sAge\_1

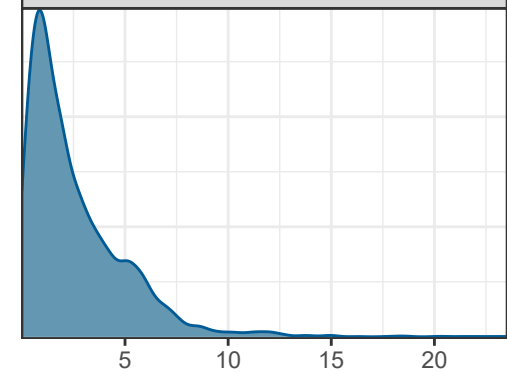

lp\_\_

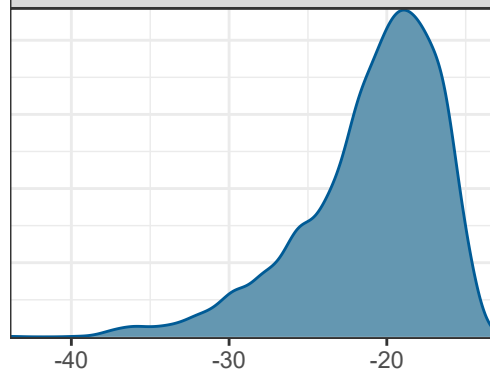

Supplement: Supplementary file 1 — Additional file 1: Figure S1. Effects of the age and the sex of the subject, and the time-period between the vaccination and the measurement on the probability of the lack of RBD-specific antibody production (titre below 1) after two doses of the Sinopharm vaccine using logistic regression model. 90% credible interval is shown for males, 28 days post second dose. Figure S2. Effects of the age and the sex of the subject on the probability of the lack of RBD-specific antibody production (titre below 1) after two doses of the Pfizer/BioNTech vaccine using logistic regression model. 90% credible interval is shown for males, 28 days post second dose. Figure S3. Sinopharm vaccine model, MCMC diagnostics: density plot for the hurdle-lognormal model. Figure S4. Sinopharm vaccine model, MCMC diagnostics: density plot for the logistic model. Figure S5. Sinopharm vaccine model, MCMC diagnostics: trace plot for the hurdle-lognormal model. Figure S6. Sinopharm vaccine model, MCMC diagnostics: trace plot for the logistic model. Figure S7. Sinopharm vaccine model, MCMC diagnostics: autocorrelation function for the hurdle-lognormal model. Figure S8. Sinopharm vaccine model, MCMC diagnostics: autocorrelation function for the logistic model. Figure S9. Sinopharm vaccine model, MCMC diagnostics: posterior predictive check for the hurdle-lognormal model. Figure S10. Sinopharm vaccine model, MCMC diagnostics: posterior predictive check for the logistic model. Figure S11. Pfizer/BioNTech vaccine model, MCMC diagnostics: density plot for the hurdle-lognormal model. Figure S12. Pfizer/BioNTech vaccine model, MCMC diagnostics: density plot for the logistic model. Figure S13. Pfizer/BioNTech vaccine model, MCMC diagnostics: trace plot for the hurdle-lognormal model. Figure S14. Pfizer/BioNTech vaccine model, MCMC diagnostics: trace plot for the logistic model. Figure S15. Pfizer/BioNTech vaccine model, MCMC diagnostics: autocorrelation function for the hurdle-lognormal model. Figure S16. Pfi [file 12879_2022_7069_MOESM1_ESM.zip › 12879_2022_7069_MOESM1_ESM/FigureS12.pdf]

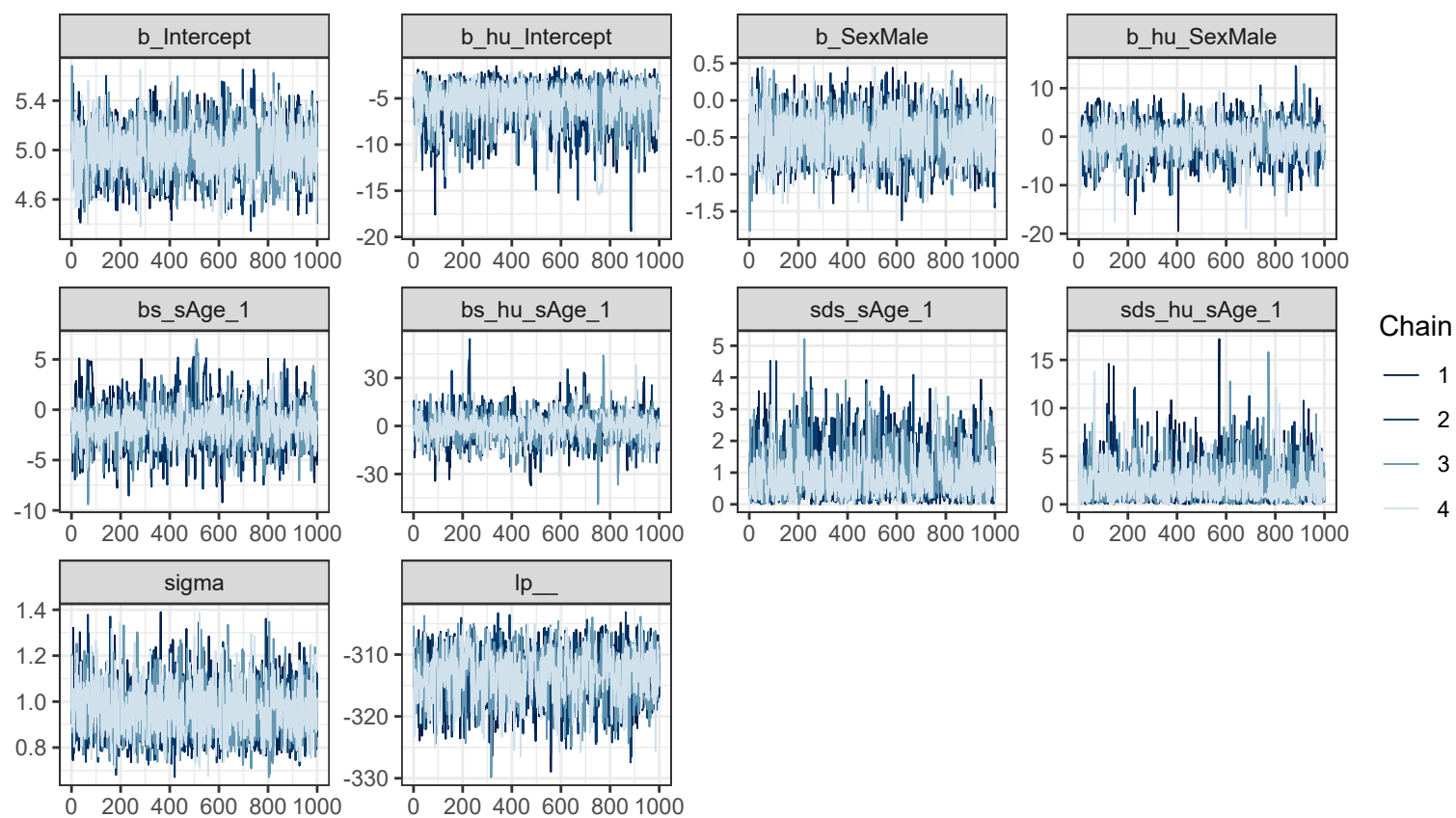

Supplement: Supplementary file 1 — Additional file 1: Figure S1. Effects of the age and the sex of the subject, and the time-period between the vaccination and the measurement on the probability of the lack of RBD-specific antibody production (titre below 1) after two doses of the Sinopharm vaccine using logistic regression model. 90% credible interval is shown for males, 28 days post second dose. Figure S2. Effects of the age and the sex of the subject on the probability of the lack of RBD-specific antibody production (titre below 1) after two doses of the Pfizer/BioNTech vaccine using logistic regression model. 90% credible interval is shown for males, 28 days post second dose. Figure S3. Sinopharm vaccine model, MCMC diagnostics: density plot for the hurdle-lognormal model. Figure S4. Sinopharm vaccine model, MCMC diagnostics: density plot for the logistic model. Figure S5. Sinopharm vaccine model, MCMC diagnostics: trace plot for the hurdle-lognormal model. Figure S6. Sinopharm vaccine model, MCMC diagnostics: trace plot for the logistic model. Figure S7. Sinopharm vaccine model, MCMC diagnostics: autocorrelation function for the hurdle-lognormal model. Figure S8. Sinopharm vaccine model, MCMC diagnostics: autocorrelation function for the logistic model. Figure S9. Sinopharm vaccine model, MCMC diagnostics: posterior predictive check for the hurdle-lognormal model. Figure S10. Sinopharm vaccine model, MCMC diagnostics: posterior predictive check for the logistic model. Figure S11. Pfizer/BioNTech vaccine model, MCMC diagnostics: density plot for the hurdle-lognormal model. Figure S12. Pfizer/BioNTech vaccine model, MCMC diagnostics: density plot for the logistic model. Figure S13. Pfizer/BioNTech vaccine model, MCMC diagnostics: trace plot for the hurdle-lognormal model. Figure S14. Pfizer/BioNTech vaccine model, MCMC diagnostics: trace plot for the logistic model. Figure S15. Pfizer/BioNTech vaccine model, MCMC diagnostics: autocorrelation function for the hurdle-lognormal model. Figure S16. Pfi [file 12879_2022_7069_MOESM1_ESM.zip › 12879_2022_7069_MOESM1_ESM/FigureS13.pdf]

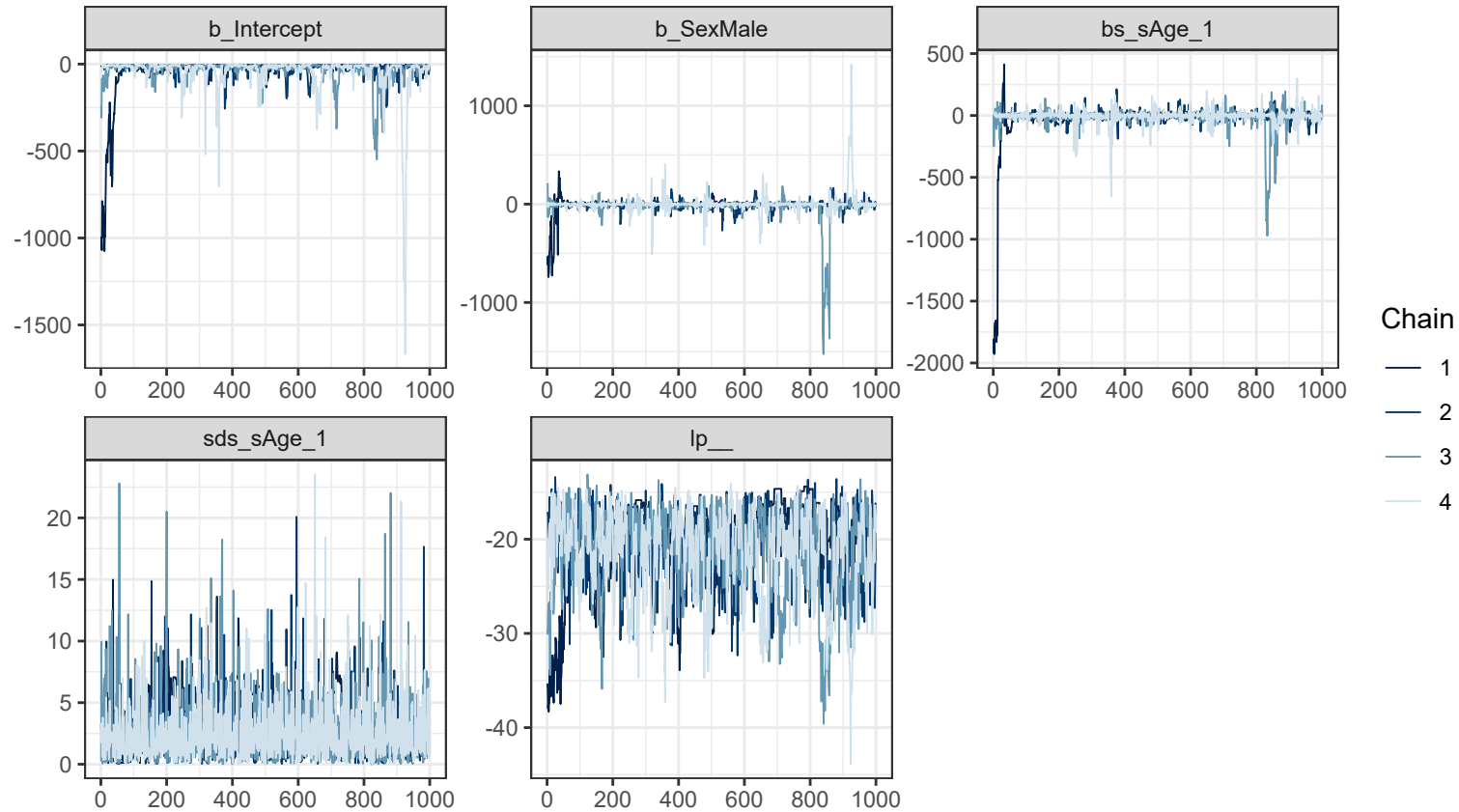

Supplement: Supplementary file 1 — Additional file 1: Figure S1. Effects of the age and the sex of the subject, and the time-period between the vaccination and the measurement on the probability of the lack of RBD-specific antibody production (titre below 1) after two doses of the Sinopharm vaccine using logistic regression model. 90% credible interval is shown for males, 28 days post second dose. Figure S2. Effects of the age and the sex of the subject on the probability of the lack of RBD-specific antibody production (titre below 1) after two doses of the Pfizer/BioNTech vaccine using logistic regression model. 90% credible interval is shown for males, 28 days post second dose. Figure S3. Sinopharm vaccine model, MCMC diagnostics: density plot for the hurdle-lognormal model. Figure S4. Sinopharm vaccine model, MCMC diagnostics: density plot for the logistic model. Figure S5. Sinopharm vaccine model, MCMC diagnostics: trace plot for the hurdle-lognormal model. Figure S6. Sinopharm vaccine model, MCMC diagnostics: trace plot for the logistic model. Figure S7. Sinopharm vaccine model, MCMC diagnostics: autocorrelation function for the hurdle-lognormal model. Figure S8. Sinopharm vaccine model, MCMC diagnostics: autocorrelation function for the logistic model. Figure S9. Sinopharm vaccine model, MCMC diagnostics: posterior predictive check for the hurdle-lognormal model. Figure S10. Sinopharm vaccine model, MCMC diagnostics: posterior predictive check for the logistic model. Figure S11. Pfizer/BioNTech vaccine model, MCMC diagnostics: density plot for the hurdle-lognormal model. Figure S12. Pfizer/BioNTech vaccine model, MCMC diagnostics: density plot for the logistic model. Figure S13. Pfizer/BioNTech vaccine model, MCMC diagnostics: trace plot for the hurdle-lognormal model. Figure S14. Pfizer/BioNTech vaccine model, MCMC diagnostics: trace plot for the logistic model. Figure S15. Pfizer/BioNTech vaccine model, MCMC diagnostics: autocorrelation function for the hurdle-lognormal model. Figure S16. Pfi [file 12879_2022_7069_MOESM1_ESM.zip › 12879_2022_7069_MOESM1_ESM/FigureS14.pdf]

Autocorrelation

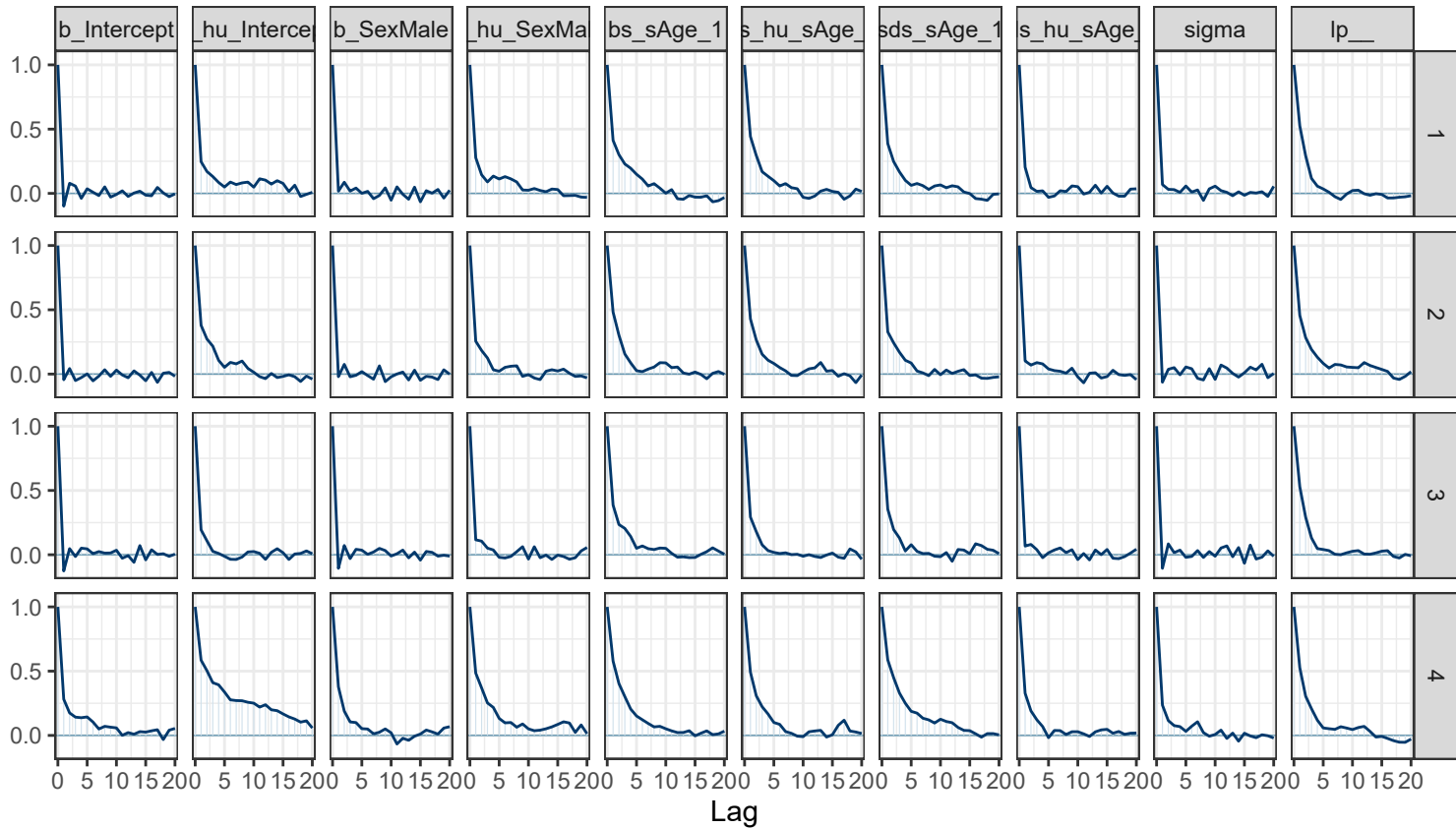

Supplement: Supplementary file 1 — Additional file 1: Figure S1. Effects of the age and the sex of the subject, and the time-period between the vaccination and the measurement on the probability of the lack of RBD-specific antibody production (titre below 1) after two doses of the Sinopharm vaccine using logistic regression model. 90% credible interval is shown for males, 28 days post second dose. Figure S2. Effects of the age and the sex of the subject on the probability of the lack of RBD-specific antibody production (titre below 1) after two doses of the Pfizer/BioNTech vaccine using logistic regression model. 90% credible interval is shown for males, 28 days post second dose. Figure S3. Sinopharm vaccine model, MCMC diagnostics: density plot for the hurdle-lognormal model. Figure S4. Sinopharm vaccine model, MCMC diagnostics: density plot for the logistic model. Figure S5. Sinopharm vaccine model, MCMC diagnostics: trace plot for the hurdle-lognormal model. Figure S6. Sinopharm vaccine model, MCMC diagnostics: trace plot for the logistic model. Figure S7. Sinopharm vaccine model, MCMC diagnostics: autocorrelation function for the hurdle-lognormal model. Figure S8. Sinopharm vaccine model, MCMC diagnostics: autocorrelation function for the logistic model. Figure S9. Sinopharm vaccine model, MCMC diagnostics: posterior predictive check for the hurdle-lognormal model. Figure S10. Sinopharm vaccine model, MCMC diagnostics: posterior predictive check for the logistic model. Figure S11. Pfizer/BioNTech vaccine model, MCMC diagnostics: density plot for the hurdle-lognormal model. Figure S12. Pfizer/BioNTech vaccine model, MCMC diagnostics: density plot for the logistic model. Figure S13. Pfizer/BioNTech vaccine model, MCMC diagnostics: trace plot for the hurdle-lognormal model. Figure S14. Pfizer/BioNTech vaccine model, MCMC diagnostics: trace plot for the logistic model. Figure S15. Pfizer/BioNTech vaccine model, MCMC diagnostics: autocorrelation function for the hurdle-lognormal model. Figure S16. Pfi [file 12879_2022_7069_MOESM1_ESM.zip › 12879_2022_7069_MOESM1_ESM/FigureS15.pdf]

Autocorrelation

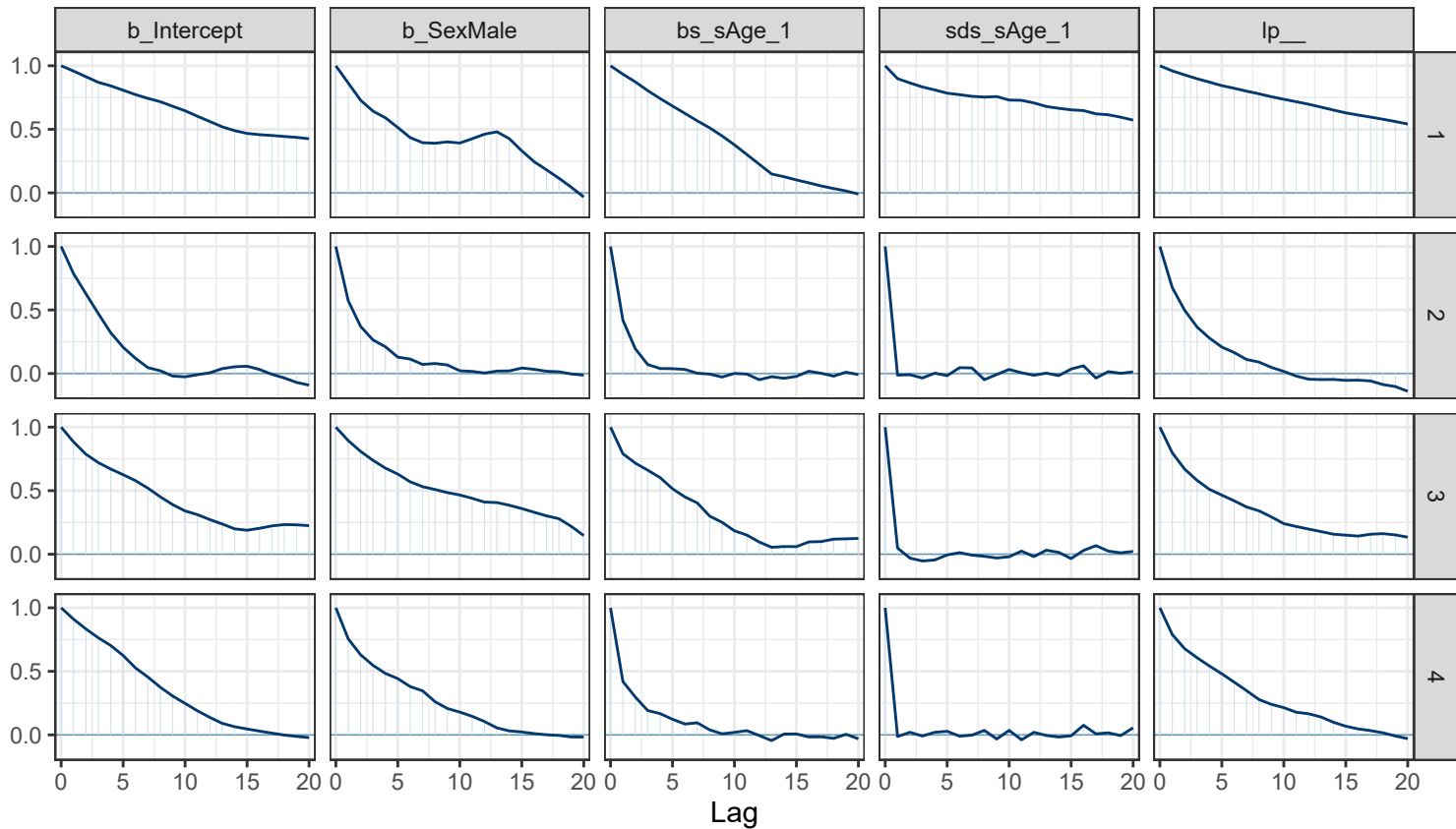

Supplement: Supplementary file 1 — Additional file 1: Figure S1. Effects of the age and the sex of the subject, and the time-period between the vaccination and the measurement on the probability of the lack of RBD-specific antibody production (titre below 1) after two doses of the Sinopharm vaccine using logistic regression model. 90% credible interval is shown for males, 28 days post second dose. Figure S2. Effects of the age and the sex of the subject on the probability of the lack of RBD-specific antibody production (titre below 1) after two doses of the Pfizer/BioNTech vaccine using logistic regression model. 90% credible interval is shown for males, 28 days post second dose. Figure S3. Sinopharm vaccine model, MCMC diagnostics: density plot for the hurdle-lognormal model. Figure S4. Sinopharm vaccine model, MCMC diagnostics: density plot for the logistic model. Figure S5. Sinopharm vaccine model, MCMC diagnostics: trace plot for the hurdle-lognormal model. Figure S6. Sinopharm vaccine model, MCMC diagnostics: trace plot for the logistic model. Figure S7. Sinopharm vaccine model, MCMC diagnostics: autocorrelation function for the hurdle-lognormal model. Figure S8. Sinopharm vaccine model, MCMC diagnostics: autocorrelation function for the logistic model. Figure S9. Sinopharm vaccine model, MCMC diagnostics: posterior predictive check for the hurdle-lognormal model. Figure S10. Sinopharm vaccine model, MCMC diagnostics: posterior predictive check for the logistic model. Figure S11. Pfizer/BioNTech vaccine model, MCMC diagnostics: density plot for the hurdle-lognormal model. Figure S12. Pfizer/BioNTech vaccine model, MCMC diagnostics: density plot for the logistic model. Figure S13. Pfizer/BioNTech vaccine model, MCMC diagnostics: trace plot for the hurdle-lognormal model. Figure S14. Pfizer/BioNTech vaccine model, MCMC diagnostics: trace plot for the logistic model. Figure S15. Pfizer/BioNTech vaccine model, MCMC diagnostics: autocorrelation function for the hurdle-lognormal model. Figure S16. Pfi [file 12879_2022_7069_MOESM1_ESM.zip › 12879_2022_7069_MOESM1_ESM/FigureS16.pdf]

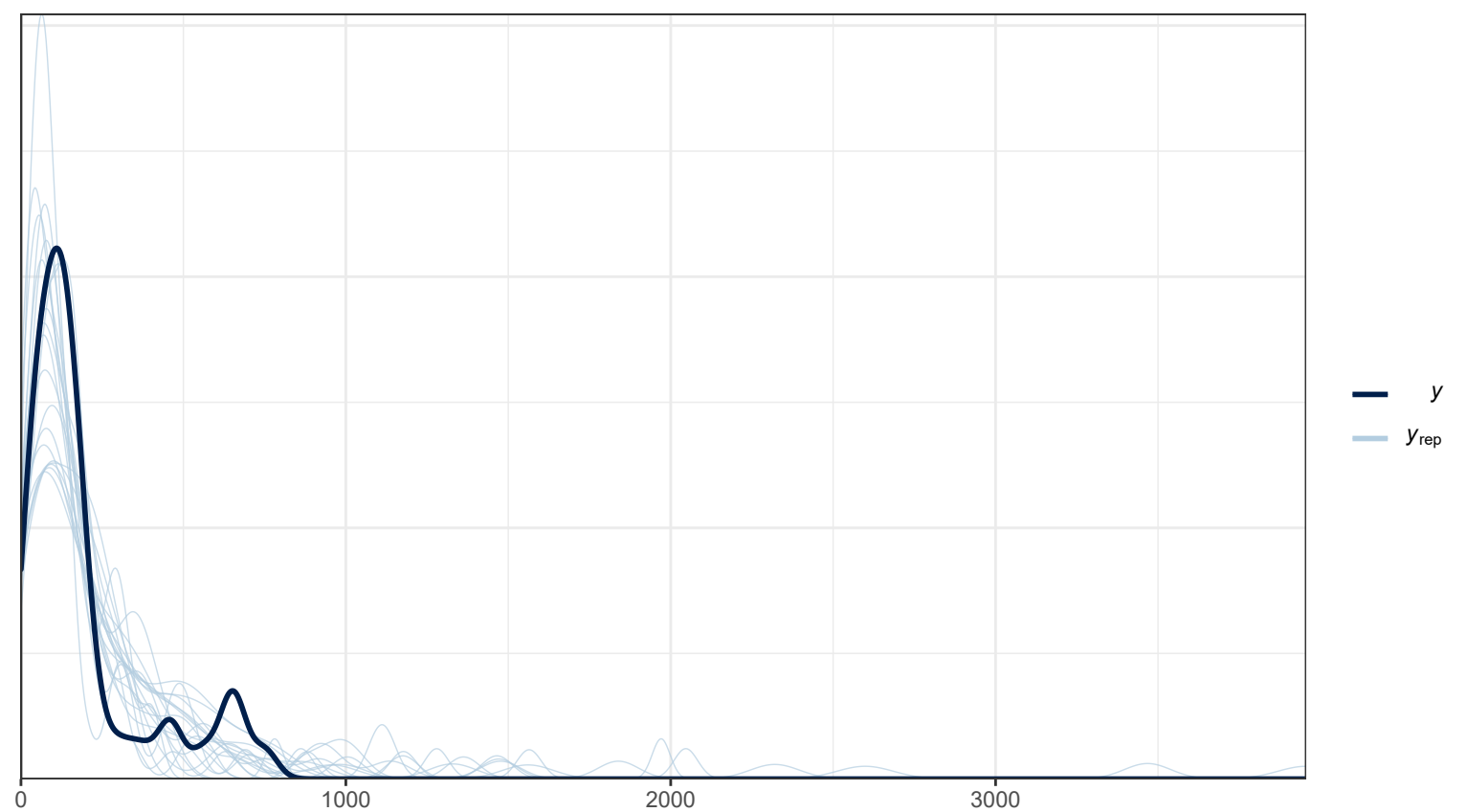

Supplement: Supplementary file 1 — Additional file 1: Figure S1. Effects of the age and the sex of the subject, and the time-period between the vaccination and the measurement on the probability of the lack of RBD-specific antibody production (titre below 1) after two doses of the Sinopharm vaccine using logistic regression model. 90% credible interval is shown for males, 28 days post second dose. Figure S2. Effects of the age and the sex of the subject on the probability of the lack of RBD-specific antibody production (titre below 1) after two doses of the Pfizer/BioNTech vaccine using logistic regression model. 90% credible interval is shown for males, 28 days post second dose. Figure S3. Sinopharm vaccine model, MCMC diagnostics: density plot for the hurdle-lognormal model. Figure S4. Sinopharm vaccine model, MCMC diagnostics: density plot for the logistic model. Figure S5. Sinopharm vaccine model, MCMC diagnostics: trace plot for the hurdle-lognormal model. Figure S6. Sinopharm vaccine model, MCMC diagnostics: trace plot for the logistic model. Figure S7. Sinopharm vaccine model, MCMC diagnostics: autocorrelation function for the hurdle-lognormal model. Figure S8. Sinopharm vaccine model, MCMC diagnostics: autocorrelation function for the logistic model. Figure S9. Sinopharm vaccine model, MCMC diagnostics: posterior predictive check for the hurdle-lognormal model. Figure S10. Sinopharm vaccine model, MCMC diagnostics: posterior predictive check for the logistic model. Figure S11. Pfizer/BioNTech vaccine model, MCMC diagnostics: density plot for the hurdle-lognormal model. Figure S12. Pfizer/BioNTech vaccine model, MCMC diagnostics: density plot for the logistic model. Figure S13. Pfizer/BioNTech vaccine model, MCMC diagnostics: trace plot for the hurdle-lognormal model. Figure S14. Pfizer/BioNTech vaccine model, MCMC diagnostics: trace plot for the logistic model. Figure S15. Pfizer/BioNTech vaccine model, MCMC diagnostics: autocorrelation function for the hurdle-lognormal model. Figure S16. Pfi [file 12879_2022_7069_MOESM1_ESM.zip › 12879_2022_7069_MOESM1_ESM/FigureS17.pdf]

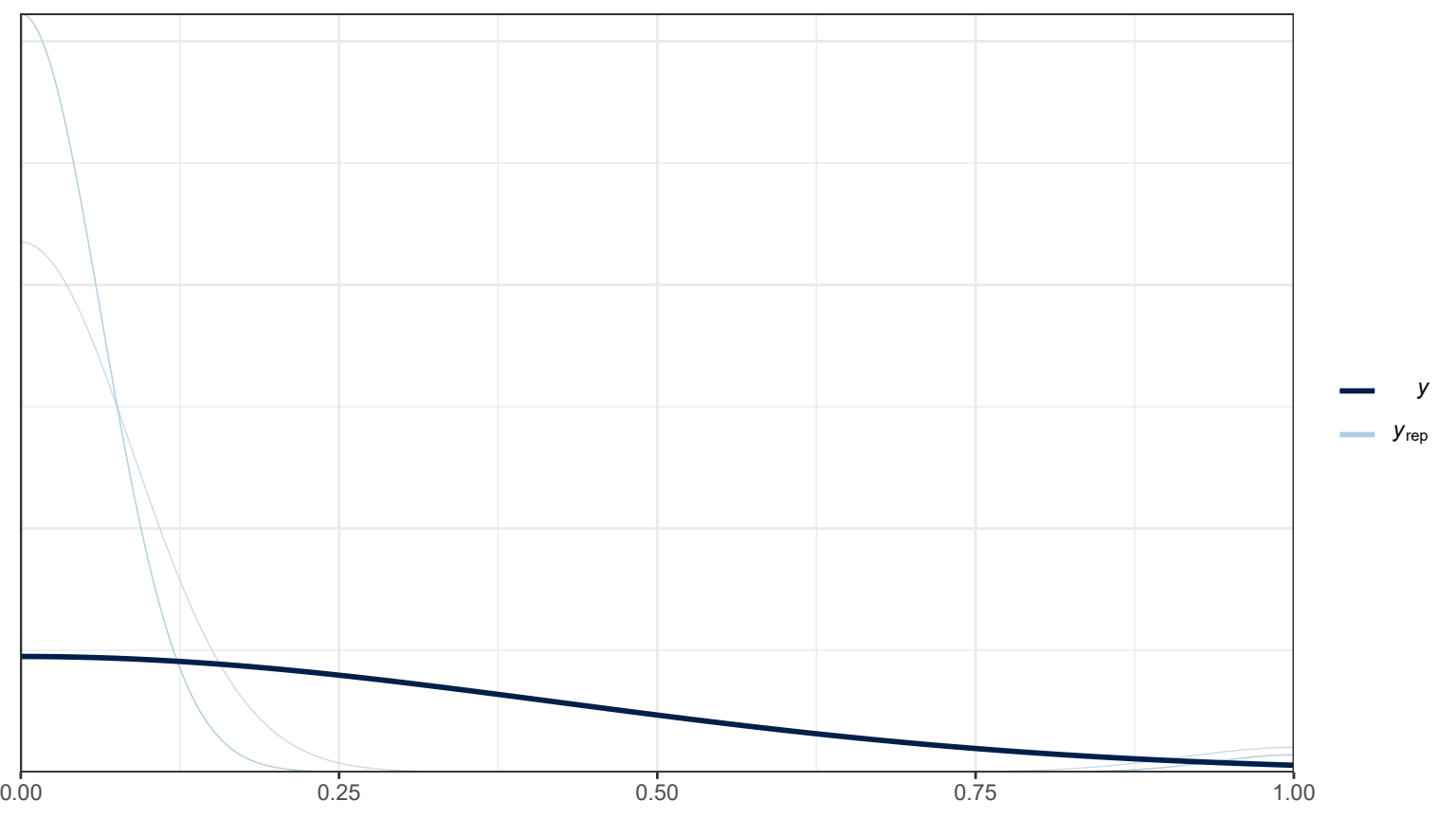

Supplement: Supplementary file 1 — Additional file 1: Figure S1. Effects of the age and the sex of the subject, and the time-period between the vaccination and the measurement on the probability of the lack of RBD-specific antibody production (titre below 1) after two doses of the Sinopharm vaccine using logistic regression model. 90% credible interval is shown for males, 28 days post second dose. Figure S2. Effects of the age and the sex of the subject on the probability of the lack of RBD-specific antibody production (titre below 1) after two doses of the Pfizer/BioNTech vaccine using logistic regression model. 90% credible interval is shown for males, 28 days post second dose. Figure S3. Sinopharm vaccine model, MCMC diagnostics: density plot for the hurdle-lognormal model. Figure S4. Sinopharm vaccine model, MCMC diagnostics: density plot for the logistic model. Figure S5. Sinopharm vaccine model, MCMC diagnostics: trace plot for the hurdle-lognormal model. Figure S6. Sinopharm vaccine model, MCMC diagnostics: trace plot for the logistic model. Figure S7. Sinopharm vaccine model, MCMC diagnostics: autocorrelation function for the hurdle-lognormal model. Figure S8. Sinopharm vaccine model, MCMC diagnostics: autocorrelation function for the logistic model. Figure S9. Sinopharm vaccine model, MCMC diagnostics: posterior predictive check for the hurdle-lognormal model. Figure S10. Sinopharm vaccine model, MCMC diagnostics: posterior predictive check for the logistic model. Figure S11. Pfizer/BioNTech vaccine model, MCMC diagnostics: density plot for the hurdle-lognormal model. Figure S12. Pfizer/BioNTech vaccine model, MCMC diagnostics: density plot for the logistic model. Figure S13. Pfizer/BioNTech vaccine model, MCMC diagnostics: trace plot for the hurdle-lognormal model. Figure S14. Pfizer/BioNTech vaccine model, MCMC diagnostics: trace plot for the logistic model. Figure S15. Pfizer/BioNTech vaccine model, MCMC diagnostics: autocorrelation function for the hurdle-lognormal model. Figure S16. Pfi [file 12879_2022_7069_MOESM1_ESM.zip › 12879_2022_7069_MOESM1_ESM/FigureS18.pdf]

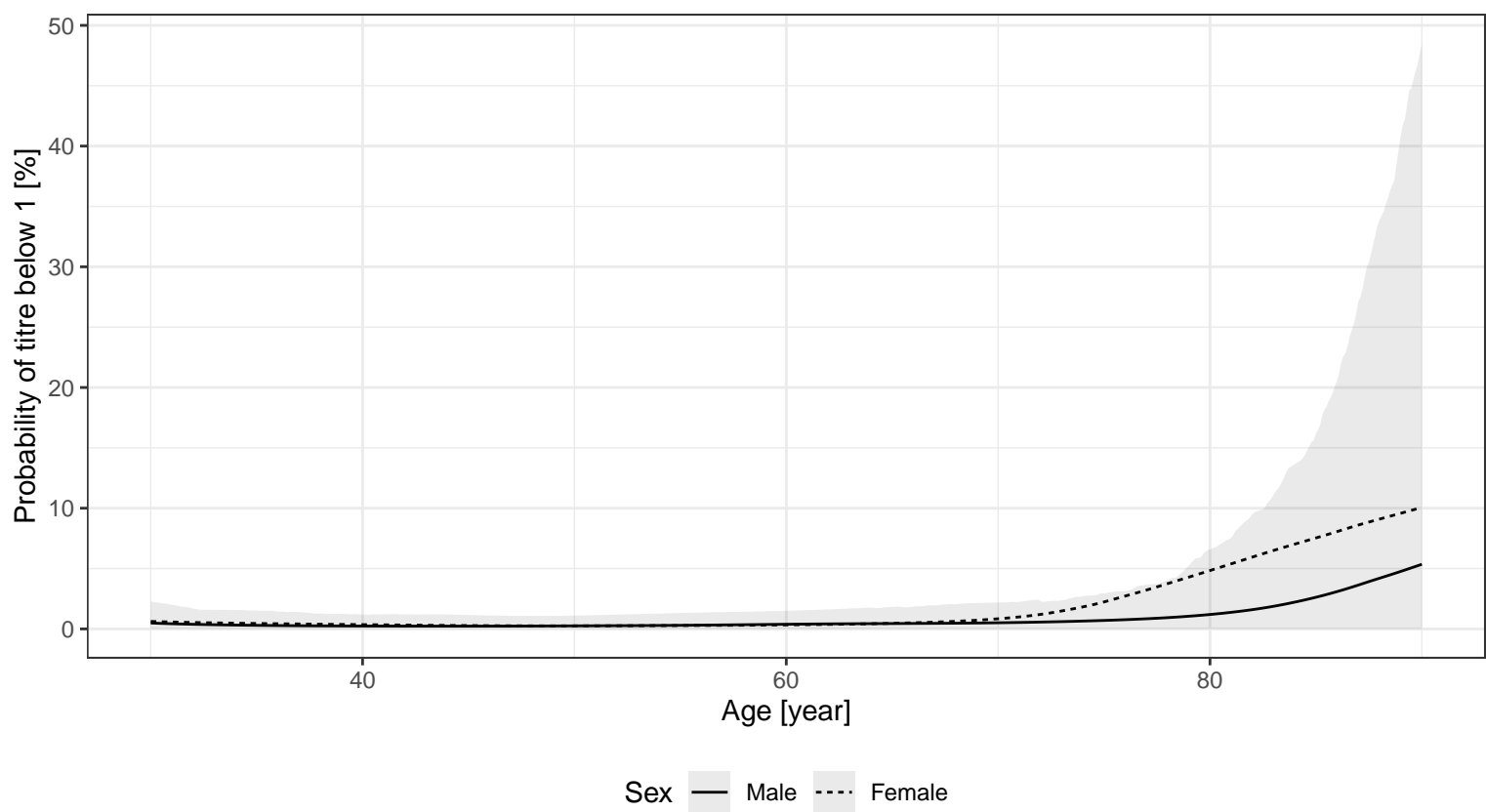

Supplement: Supplementary file 1 — Additional file 1: Figure S1. Effects of the age and the sex of the subject, and the time-period between the vaccination and the measurement on the probability of the lack of RBD-specific antibody production (titre below 1) after two doses of the Sinopharm vaccine using logistic regression model. 90% credible interval is shown for males, 28 days post second dose. Figure S2. Effects of the age and the sex of the subject on the probability of the lack of RBD-specific antibody production (titre below 1) after two doses of the Pfizer/BioNTech vaccine using logistic regression model. 90% credible interval is shown for males, 28 days post second dose. Figure S3. Sinopharm vaccine model, MCMC diagnostics: density plot for the hurdle-lognormal model. Figure S4. Sinopharm vaccine model, MCMC diagnostics: density plot for the logistic model. Figure S5. Sinopharm vaccine model, MCMC diagnostics: trace plot for the hurdle-lognormal model. Figure S6. Sinopharm vaccine model, MCMC diagnostics: trace plot for the logistic model. Figure S7. Sinopharm vaccine model, MCMC diagnostics: autocorrelation function for the hurdle-lognormal model. Figure S8. Sinopharm vaccine model, MCMC diagnostics: autocorrelation function for the logistic model. Figure S9. Sinopharm vaccine model, MCMC diagnostics: posterior predictive check for the hurdle-lognormal model. Figure S10. Sinopharm vaccine model, MCMC diagnostics: posterior predictive check for the logistic model. Figure S11. Pfizer/BioNTech vaccine model, MCMC diagnostics: density plot for the hurdle-lognormal model. Figure S12. Pfizer/BioNTech vaccine model, MCMC diagnostics: density plot for the logistic model. Figure S13. Pfizer/BioNTech vaccine model, MCMC diagnostics: trace plot for the hurdle-lognormal model. Figure S14. Pfizer/BioNTech vaccine model, MCMC diagnostics: trace plot for the logistic model. Figure S15. Pfizer/BioNTech vaccine model, MCMC diagnostics: autocorrelation function for the hurdle-lognormal model. Figure S16. Pfi [file 12879_2022_7069_MOESM1_ESM.zip › 12879_2022_7069_MOESM1_ESM/FigureS2.pdf]

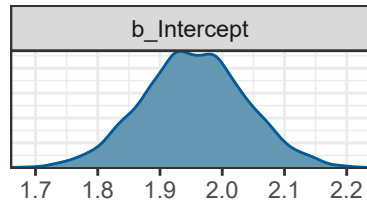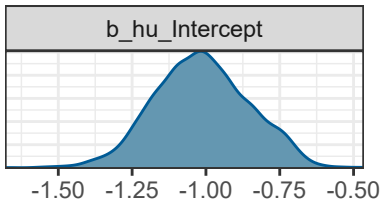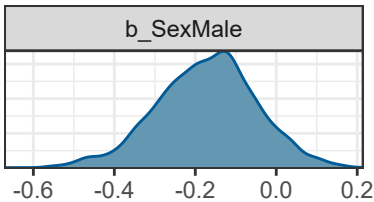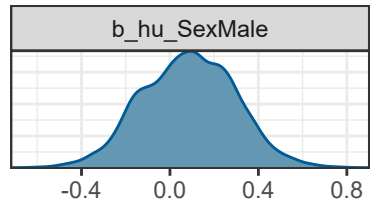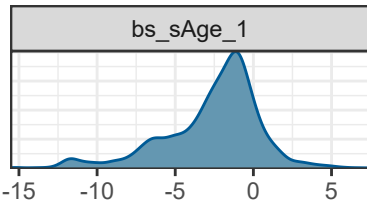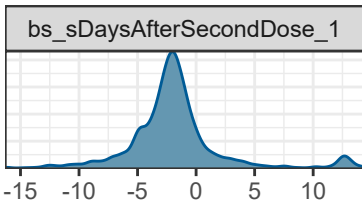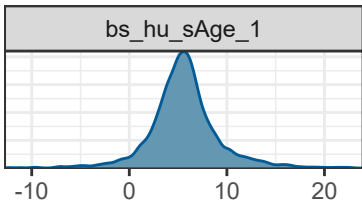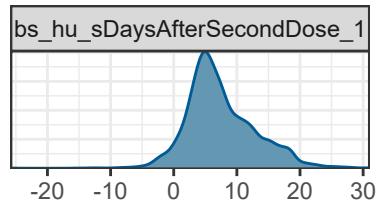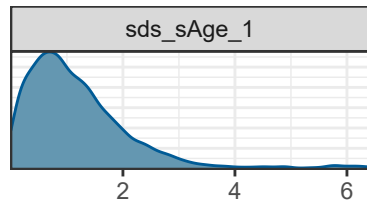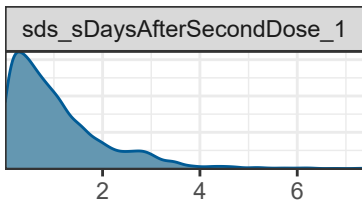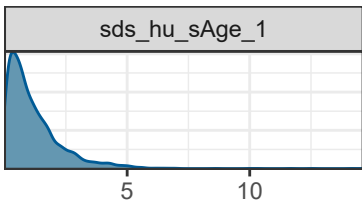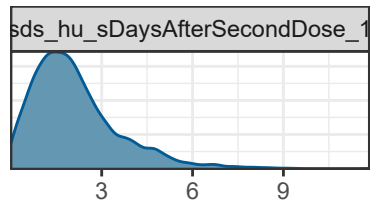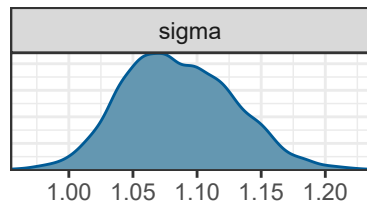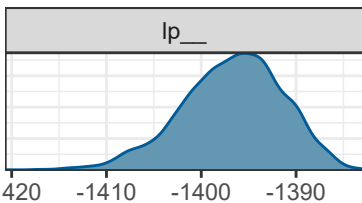

Supplement: Supplementary file 1 — Additional file 1: Figure S1. Effects of the age and the sex of the subject, and the time-period between the vaccination and the measurement on the probability of the lack of RBD-specific antibody production (titre below 1) after two doses of the Sinopharm vaccine using logistic regression model. 90% credible interval is shown for males, 28 days post second dose. Figure S2. Effects of the age and the sex of the subject on the probability of the lack of RBD-specific antibody production (titre below 1) after two doses of the Pfizer/BioNTech vaccine using logistic regression model. 90% credible interval is shown for males, 28 days post second dose. Figure S3. Sinopharm vaccine model, MCMC diagnostics: density plot for the hurdle-lognormal model. Figure S4. Sinopharm vaccine model, MCMC diagnostics: density plot for the logistic model. Figure S5. Sinopharm vaccine model, MCMC diagnostics: trace plot for the hurdle-lognormal model. Figure S6. Sinopharm vaccine model, MCMC diagnostics: trace plot for the logistic model. Figure S7. Sinopharm vaccine model, MCMC diagnostics: autocorrelation function for the hurdle-lognormal model. Figure S8. Sinopharm vaccine model, MCMC diagnostics: autocorrelation function for the logistic model. Figure S9. Sinopharm vaccine model, MCMC diagnostics: posterior predictive check for the hurdle-lognormal model. Figure S10. Sinopharm vaccine model, MCMC diagnostics: posterior predictive check for the logistic model. Figure S11. Pfizer/BioNTech vaccine model, MCMC diagnostics: density plot for the hurdle-lognormal model. Figure S12. Pfizer/BioNTech vaccine model, MCMC diagnostics: density plot for the logistic model. Figure S13. Pfizer/BioNTech vaccine model, MCMC diagnostics: trace plot for the hurdle-lognormal model. Figure S14. Pfizer/BioNTech vaccine model, MCMC diagnostics: trace plot for the logistic model. Figure S15. Pfizer/BioNTech vaccine model, MCMC diagnostics: autocorrelation function for the hurdle-lognormal model. Figure S16. Pfi [file 12879_2022_7069_MOESM1_ESM.zip › 12879_2022_7069_MOESM1_ESM/FigureS3.pdf]

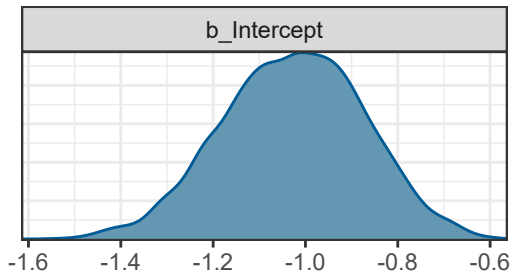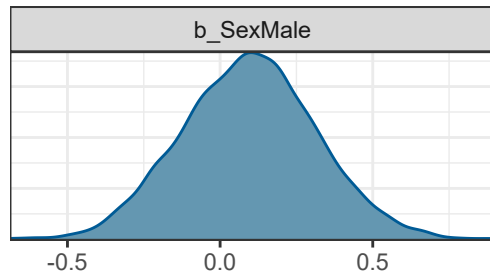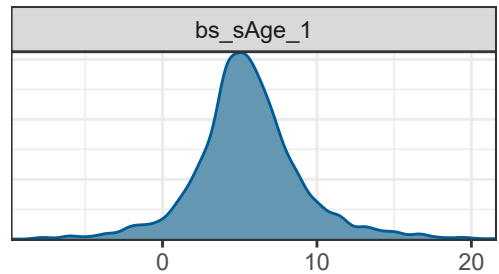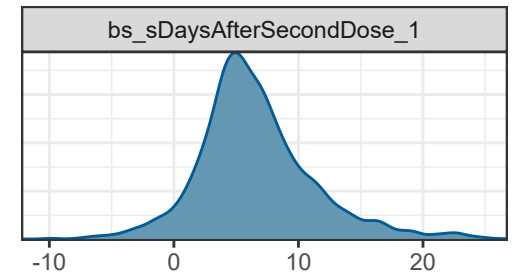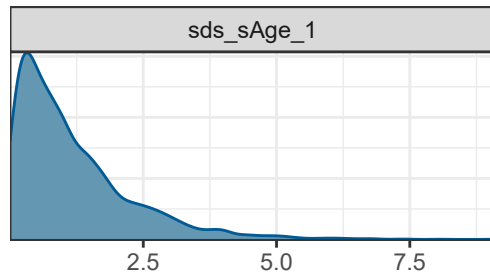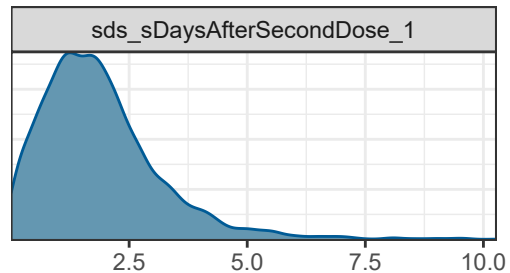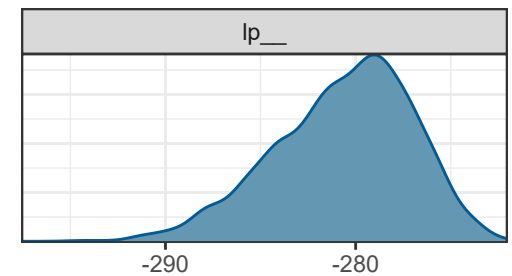

Supplement: Supplementary file 1 — Additional file 1: Figure S1. Effects of the age and the sex of the subject, and the time-period between the vaccination and the measurement on the probability of the lack of RBD-specific antibody production (titre below 1) after two doses of the Sinopharm vaccine using logistic regression model. 90% credible interval is shown for males, 28 days post second dose. Figure S2. Effects of the age and the sex of the subject on the probability of the lack of RBD-specific antibody production (titre below 1) after two doses of the Pfizer/BioNTech vaccine using logistic regression model. 90% credible interval is shown for males, 28 days post second dose. Figure S3. Sinopharm vaccine model, MCMC diagnostics: density plot for the hurdle-lognormal model. Figure S4. Sinopharm vaccine model, MCMC diagnostics: density plot for the logistic model. Figure S5. Sinopharm vaccine model, MCMC diagnostics: trace plot for the hurdle-lognormal model. Figure S6. Sinopharm vaccine model, MCMC diagnostics: trace plot for the logistic model. Figure S7. Sinopharm vaccine model, MCMC diagnostics: autocorrelation function for the hurdle-lognormal model. Figure S8. Sinopharm vaccine model, MCMC diagnostics: autocorrelation function for the logistic model. Figure S9. Sinopharm vaccine model, MCMC diagnostics: posterior predictive check for the hurdle-lognormal model. Figure S10. Sinopharm vaccine model, MCMC diagnostics: posterior predictive check for the logistic model. Figure S11. Pfizer/BioNTech vaccine model, MCMC diagnostics: density plot for the hurdle-lognormal model. Figure S12. Pfizer/BioNTech vaccine model, MCMC diagnostics: density plot for the logistic model. Figure S13. Pfizer/BioNTech vaccine model, MCMC diagnostics: trace plot for the hurdle-lognormal model. Figure S14. Pfizer/BioNTech vaccine model, MCMC diagnostics: trace plot for the logistic model. Figure S15. Pfizer/BioNTech vaccine model, MCMC diagnostics: autocorrelation function for the hurdle-lognormal model. Figure S16. Pfi [file 12879_2022_7069_MOESM1_ESM.zip › 12879_2022_7069_MOESM1_ESM/FigureS4.pdf]

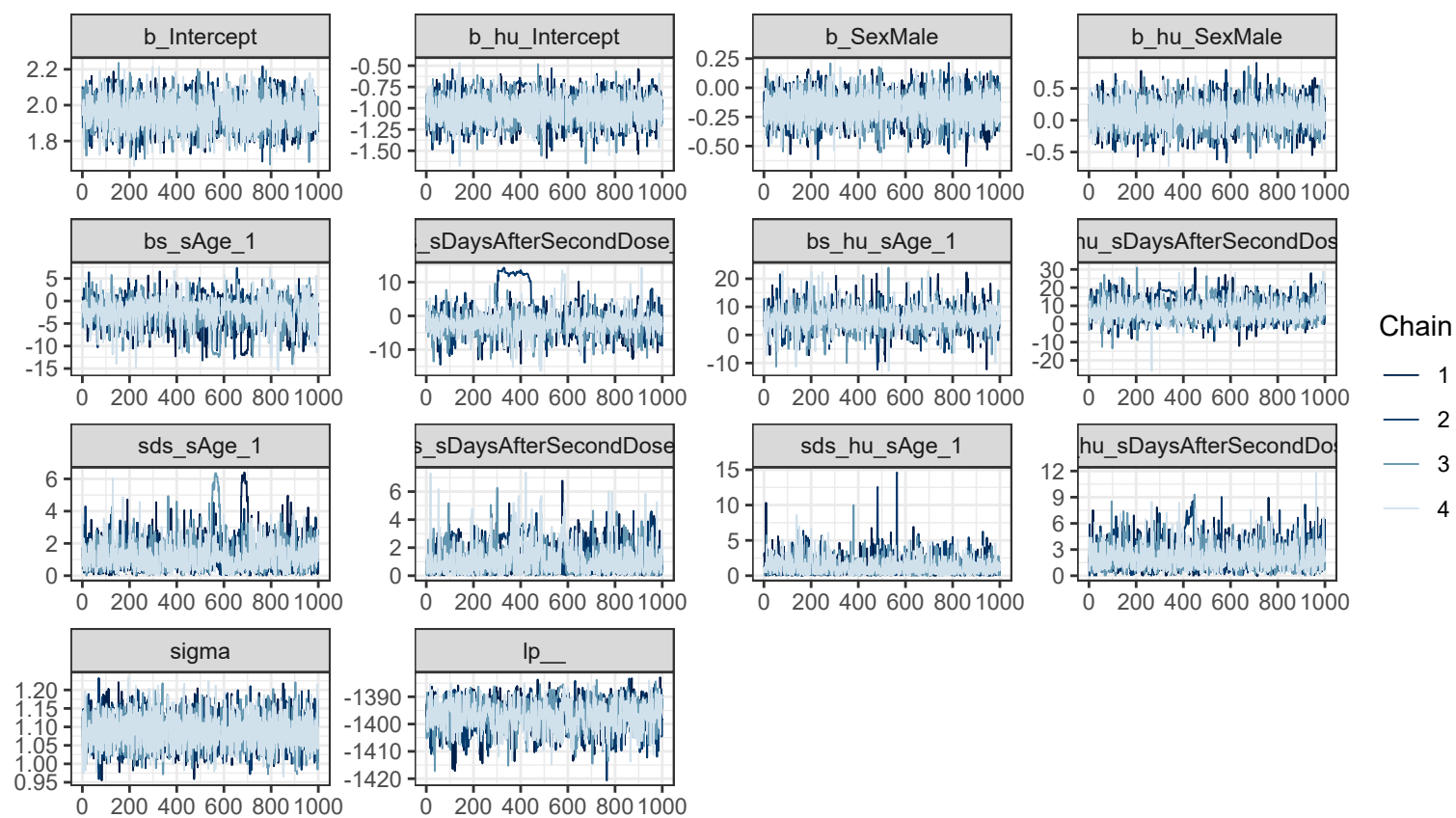

Supplement: Supplementary file 1 — Additional file 1: Figure S1. Effects of the age and the sex of the subject, and the time-period between the vaccination and the measurement on the probability of the lack of RBD-specific antibody production (titre below 1) after two doses of the Sinopharm vaccine using logistic regression model. 90% credible interval is shown for males, 28 days post second dose. Figure S2. Effects of the age and the sex of the subject on the probability of the lack of RBD-specific antibody production (titre below 1) after two doses of the Pfizer/BioNTech vaccine using logistic regression model. 90% credible interval is shown for males, 28 days post second dose. Figure S3. Sinopharm vaccine model, MCMC diagnostics: density plot for the hurdle-lognormal model. Figure S4. Sinopharm vaccine model, MCMC diagnostics: density plot for the logistic model. Figure S5. Sinopharm vaccine model, MCMC diagnostics: trace plot for the hurdle-lognormal model. Figure S6. Sinopharm vaccine model, MCMC diagnostics: trace plot for the logistic model. Figure S7. Sinopharm vaccine model, MCMC diagnostics: autocorrelation function for the hurdle-lognormal model. Figure S8. Sinopharm vaccine model, MCMC diagnostics: autocorrelation function for the logistic model. Figure S9. Sinopharm vaccine model, MCMC diagnostics: posterior predictive check for the hurdle-lognormal model. Figure S10. Sinopharm vaccine model, MCMC diagnostics: posterior predictive check for the logistic model. Figure S11. Pfizer/BioNTech vaccine model, MCMC diagnostics: density plot for the hurdle-lognormal model. Figure S12. Pfizer/BioNTech vaccine model, MCMC diagnostics: density plot for the logistic model. Figure S13. Pfizer/BioNTech vaccine model, MCMC diagnostics: trace plot for the hurdle-lognormal model. Figure S14. Pfizer/BioNTech vaccine model, MCMC diagnostics: trace plot for the logistic model. Figure S15. Pfizer/BioNTech vaccine model, MCMC diagnostics: autocorrelation function for the hurdle-lognormal model. Figure S16. Pfi [file 12879_2022_7069_MOESM1_ESM.zip › 12879_2022_7069_MOESM1_ESM/FigureS5.pdf]

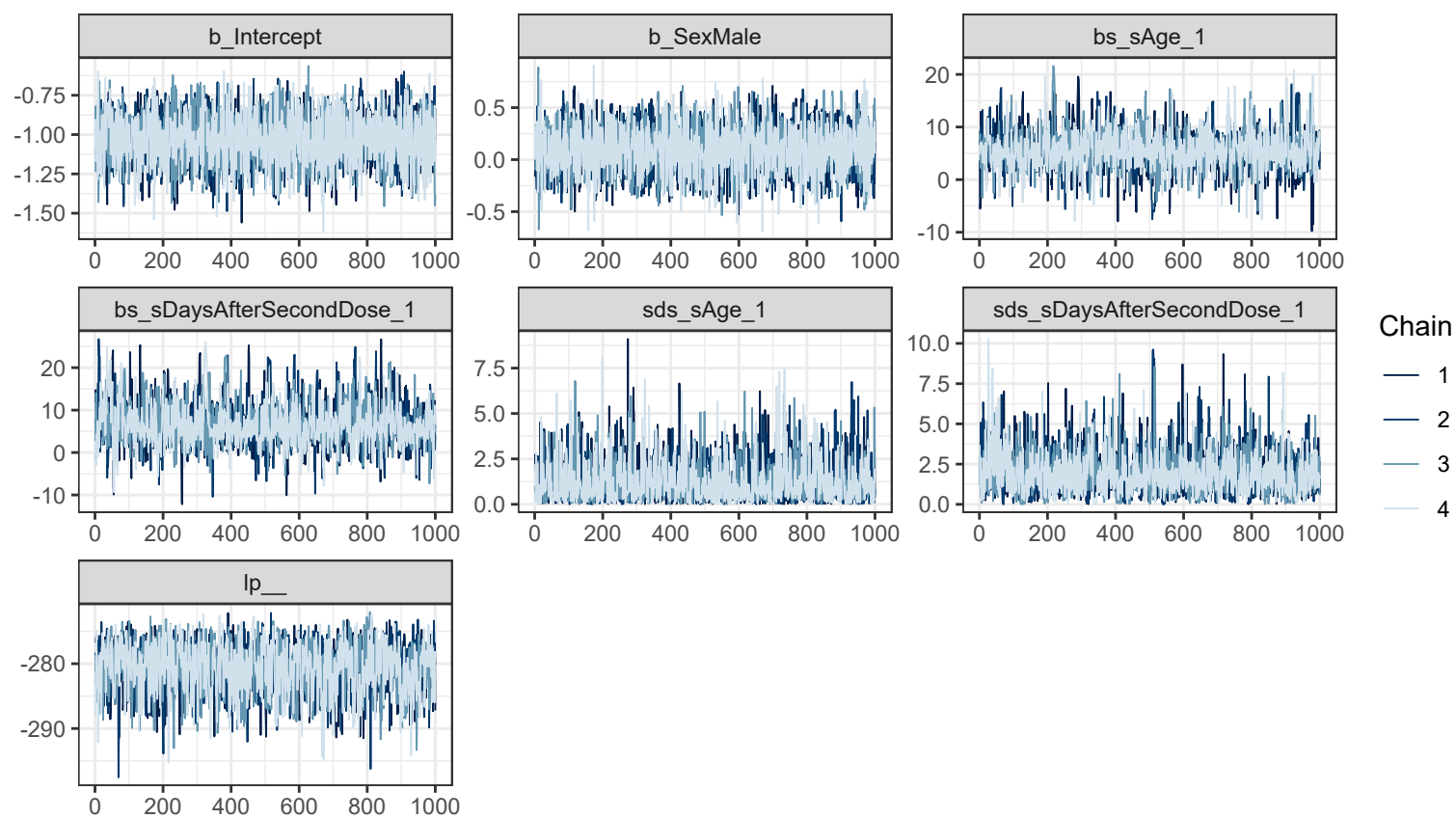

Supplement: Supplementary file 1 — Additional file 1: Figure S1. Effects of the age and the sex of the subject, and the time-period between the vaccination and the measurement on the probability of the lack of RBD-specific antibody production (titre below 1) after two doses of the Sinopharm vaccine using logistic regression model. 90% credible interval is shown for males, 28 days post second dose. Figure S2. Effects of the age and the sex of the subject on the probability of the lack of RBD-specific antibody production (titre below 1) after two doses of the Pfizer/BioNTech vaccine using logistic regression model. 90% credible interval is shown for males, 28 days post second dose. Figure S3. Sinopharm vaccine model, MCMC diagnostics: density plot for the hurdle-lognormal model. Figure S4. Sinopharm vaccine model, MCMC diagnostics: density plot for the logistic model. Figure S5. Sinopharm vaccine model, MCMC diagnostics: trace plot for the hurdle-lognormal model. Figure S6. Sinopharm vaccine model, MCMC diagnostics: trace plot for the logistic model. Figure S7. Sinopharm vaccine model, MCMC diagnostics: autocorrelation function for the hurdle-lognormal model. Figure S8. Sinopharm vaccine model, MCMC diagnostics: autocorrelation function for the logistic model. Figure S9. Sinopharm vaccine model, MCMC diagnostics: posterior predictive check for the hurdle-lognormal model. Figure S10. Sinopharm vaccine model, MCMC diagnostics: posterior predictive check for the logistic model. Figure S11. Pfizer/BioNTech vaccine model, MCMC diagnostics: density plot for the hurdle-lognormal model. Figure S12. Pfizer/BioNTech vaccine model, MCMC diagnostics: density plot for the logistic model. Figure S13. Pfizer/BioNTech vaccine model, MCMC diagnostics: trace plot for the hurdle-lognormal model. Figure S14. Pfizer/BioNTech vaccine model, MCMC diagnostics: trace plot for the logistic model. Figure S15. Pfizer/BioNTech vaccine model, MCMC diagnostics: autocorrelation function for the hurdle-lognormal model. Figure S16. Pfi [file 12879_2022_7069_MOESM1_ESM.zip › 12879_2022_7069_MOESM1_ESM/FigureS6.pdf]

Autocorrelation

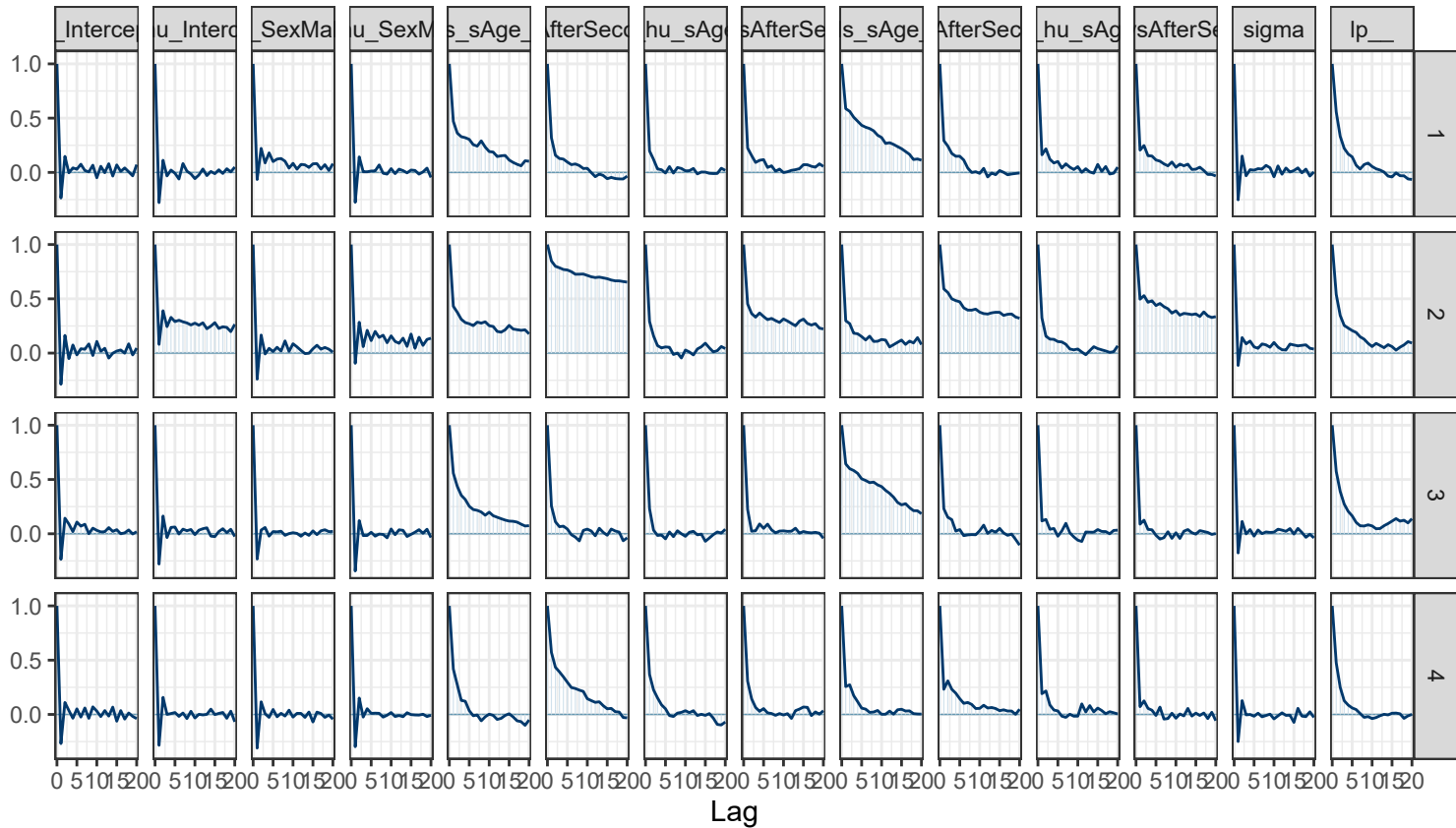

Supplement: Supplementary file 1 — Additional file 1: Figure S1. Effects of the age and the sex of the subject, and the time-period between the vaccination and the measurement on the probability of the lack of RBD-specific antibody production (titre below 1) after two doses of the Sinopharm vaccine using logistic regression model. 90% credible interval is shown for males, 28 days post second dose. Figure S2. Effects of the age and the sex of the subject on the probability of the lack of RBD-specific antibody production (titre below 1) after two doses of the Pfizer/BioNTech vaccine using logistic regression model. 90% credible interval is shown for males, 28 days post second dose. Figure S3. Sinopharm vaccine model, MCMC diagnostics: density plot for the hurdle-lognormal model. Figure S4. Sinopharm vaccine model, MCMC diagnostics: density plot for the logistic model. Figure S5. Sinopharm vaccine model, MCMC diagnostics: trace plot for the hurdle-lognormal model. Figure S6. Sinopharm vaccine model, MCMC diagnostics: trace plot for the logistic model. Figure S7. Sinopharm vaccine model, MCMC diagnostics: autocorrelation function for the hurdle-lognormal model. Figure S8. Sinopharm vaccine model, MCMC diagnostics: autocorrelation function for the logistic model. Figure S9. Sinopharm vaccine model, MCMC diagnostics: posterior predictive check for the hurdle-lognormal model. Figure S10. Sinopharm vaccine model, MCMC diagnostics: posterior predictive check for the logistic model. Figure S11. Pfizer/BioNTech vaccine model, MCMC diagnostics: density plot for the hurdle-lognormal model. Figure S12. Pfizer/BioNTech vaccine model, MCMC diagnostics: density plot for the logistic model. Figure S13. Pfizer/BioNTech vaccine model, MCMC diagnostics: trace plot for the hurdle-lognormal model. Figure S14. Pfizer/BioNTech vaccine model, MCMC diagnostics: trace plot for the logistic model. Figure S15. Pfizer/BioNTech vaccine model, MCMC diagnostics: autocorrelation function for the hurdle-lognormal model. Figure S16. Pfi [file 12879_2022_7069_MOESM1_ESM.zip › 12879_2022_7069_MOESM1_ESM/FigureS7.pdf]

Autocorrelation

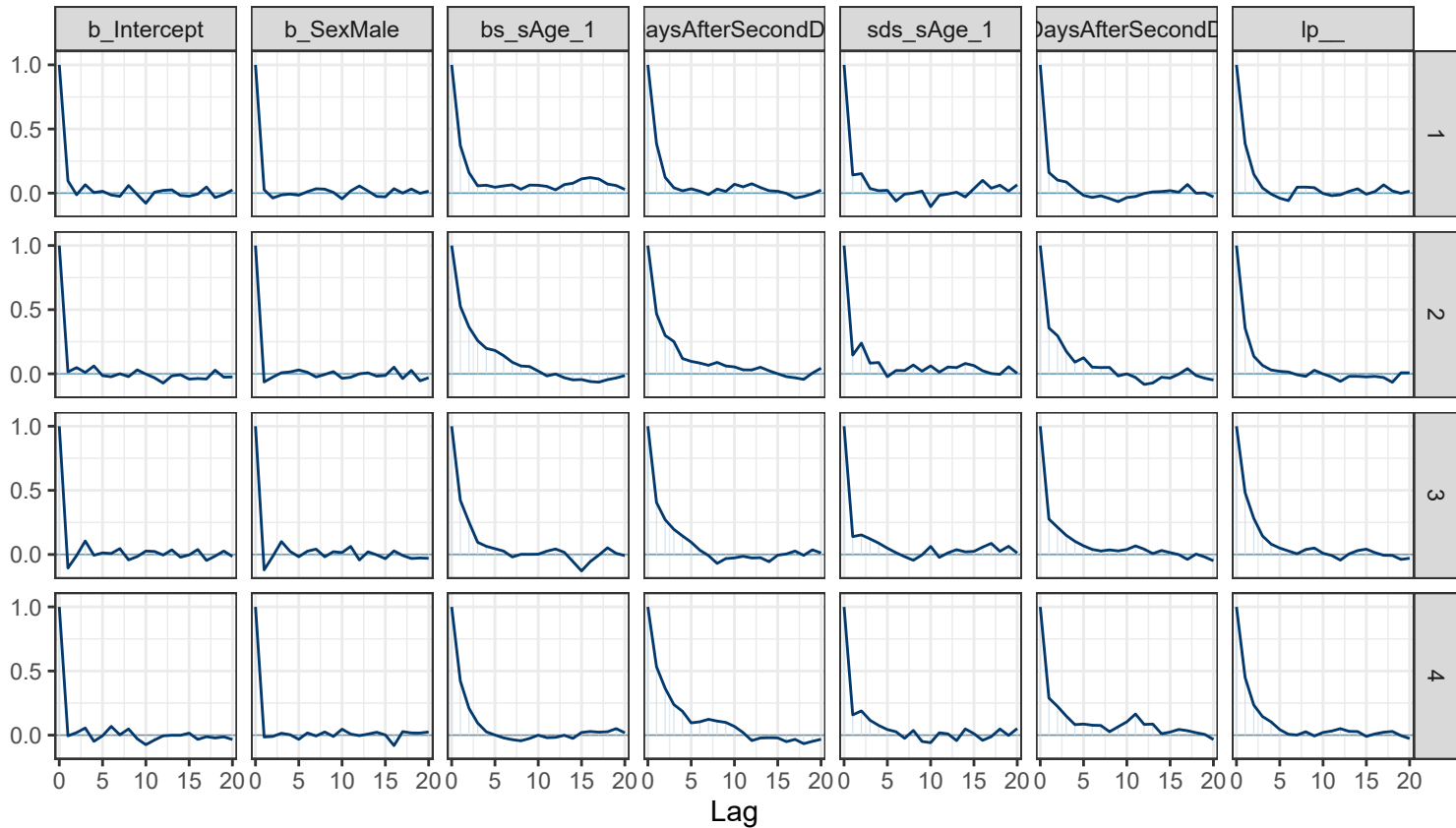

Supplement: Supplementary file 1 — Additional file 1: Figure S1. Effects of the age and the sex of the subject, and the time-period between the vaccination and the measurement on the probability of the lack of RBD-specific antibody production (titre below 1) after two doses of the Sinopharm vaccine using logistic regression model. 90% credible interval is shown for males, 28 days post second dose. Figure S2. Effects of the age and the sex of the subject on the probability of the lack of RBD-specific antibody production (titre below 1) after two doses of the Pfizer/BioNTech vaccine using logistic regression model. 90% credible interval is shown for males, 28 days post second dose. Figure S3. Sinopharm vaccine model, MCMC diagnostics: density plot for the hurdle-lognormal model. Figure S4. Sinopharm vaccine model, MCMC diagnostics: density plot for the logistic model. Figure S5. Sinopharm vaccine model, MCMC diagnostics: trace plot for the hurdle-lognormal model. Figure S6. Sinopharm vaccine model, MCMC diagnostics: trace plot for the logistic model. Figure S7. Sinopharm vaccine model, MCMC diagnostics: autocorrelation function for the hurdle-lognormal model. Figure S8. Sinopharm vaccine model, MCMC diagnostics: autocorrelation function for the logistic model. Figure S9. Sinopharm vaccine model, MCMC diagnostics: posterior predictive check for the hurdle-lognormal model. Figure S10. Sinopharm vaccine model, MCMC diagnostics: posterior predictive check for the logistic model. Figure S11. Pfizer/BioNTech vaccine model, MCMC diagnostics: density plot for the hurdle-lognormal model. Figure S12. Pfizer/BioNTech vaccine model, MCMC diagnostics: density plot for the logistic model. Figure S13. Pfizer/BioNTech vaccine model, MCMC diagnostics: trace plot for the hurdle-lognormal model. Figure S14. Pfizer/BioNTech vaccine model, MCMC diagnostics: trace plot for the logistic model. Figure S15. Pfizer/BioNTech vaccine model, MCMC diagnostics: autocorrelation function for the hurdle-lognormal model. Figure S16. Pfi [file 12879_2022_7069_MOESM1_ESM.zip › 12879_2022_7069_MOESM1_ESM/FigureS8.pdf]

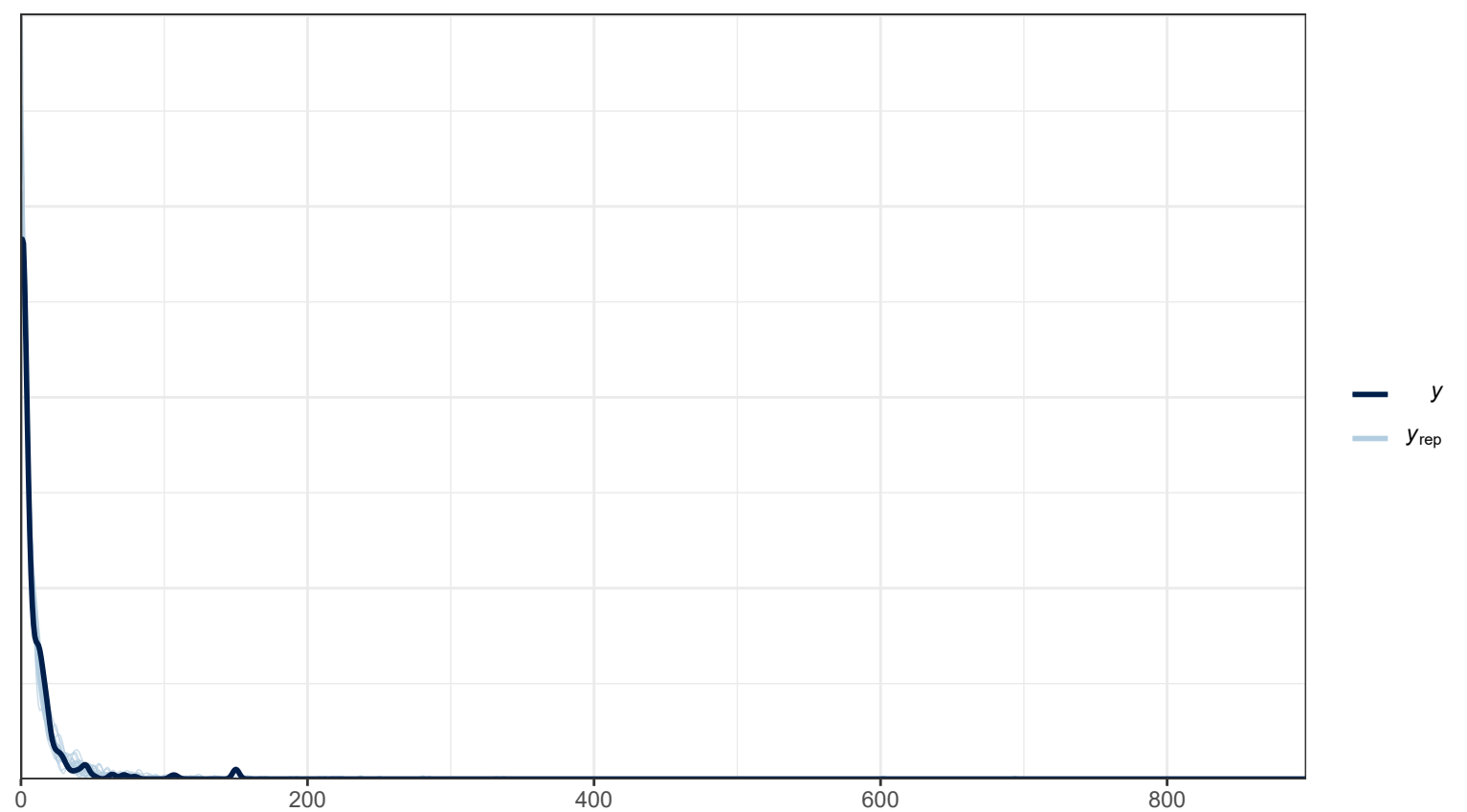

Supplement: Supplementary file 1 — Additional file 1: Figure S1. Effects of the age and the sex of the subject, and the time-period between the vaccination and the measurement on the probability of the lack of RBD-specific antibody production (titre below 1) after two doses of the Sinopharm vaccine using logistic regression model. 90% credible interval is shown for males, 28 days post second dose. Figure S2. Effects of the age and the sex of the subject on the probability of the lack of RBD-specific antibody production (titre below 1) after two doses of the Pfizer/BioNTech vaccine using logistic regression model. 90% credible interval is shown for males, 28 days post second dose. Figure S3. Sinopharm vaccine model, MCMC diagnostics: density plot for the hurdle-lognormal model. Figure S4. Sinopharm vaccine model, MCMC diagnostics: density plot for the logistic model. Figure S5. Sinopharm vaccine model, MCMC diagnostics: trace plot for the hurdle-lognormal model. Figure S6. Sinopharm vaccine model, MCMC diagnostics: trace plot for the logistic model. Figure S7. Sinopharm vaccine model, MCMC diagnostics: autocorrelation function for the hurdle-lognormal model. Figure S8. Sinopharm vaccine model, MCMC diagnostics: autocorrelation function for the logistic model. Figure S9. Sinopharm vaccine model, MCMC diagnostics: posterior predictive check for the hurdle-lognormal model. Figure S10. Sinopharm vaccine model, MCMC diagnostics: posterior predictive check for the logistic model. Figure S11. Pfizer/BioNTech vaccine model, MCMC diagnostics: density plot for the hurdle-lognormal model. Figure S12. Pfizer/BioNTech vaccine model, MCMC diagnostics: density plot for the logistic model. Figure S13. Pfizer/BioNTech vaccine model, MCMC diagnostics: trace plot for the hurdle-lognormal model. Figure S14. Pfizer/BioNTech vaccine model, MCMC diagnostics: trace plot for the logistic model. Figure S15. Pfizer/BioNTech vaccine model, MCMC diagnostics: autocorrelation function for the hurdle-lognormal model. Figure S16. Pfi [file 12879_2022_7069_MOESM1_ESM.zip › 12879_2022_7069_MOESM1_ESM/FigureS9.pdf]
